# Identification, Quantification, and Characterization of Microplastics in Skincare and Treatment Creams: A Potential Health Concern Related to the Exposure Pathway

**DOI:** 10.3390/jox16010037

**Published:** 2026-02-22

**Authors:** Raluca Maria Stirbescu, Cristiana Radulescu, Raluca Maria Bucur (Popa), Andreea Laura Banica, Ioan Alin Bucurica, Ioana Daniela Dulama

**Affiliations:** 1 Institute of Multidisciplinary Research for Science and Technology, Valahia University of Targoviste, 130004 Targoviste, Romania; stirbescu.raluca@icstm.ro (R.M.S.); banica.andreea@icstm.ro (A.L.B.); bucurica_alin@icstm.ro (I.A.B.); dulama.ioana@icstm.ro (I.D.D.); 2 Faculty of Sciences and Arts, Valahia University of Targoviste, 13 Sinaia Alley, 130004 Targoviste, Romania; 3 Doctoral School Chemical Engineering and Biotechnology, National University of Science and Technology Politehnica of Bucharest, 060042 Bucharest, Romania; ralucamaria.bucur@gmail.com; 4 Academy of Romanian Scientists, 3 Ilfov, 050044 Bucharest, Romania

**Keywords:** microplastic, cosmetic formulation, morphology, micro-FTIR, hidden risk, exposure pathway

## Abstract

This research aimed to quantify and investigate the morphology of microplastics in skincare and treatment creams related to their chemical composition and the potential risks to human health associated with exposure to microplastics by dermal contact. A total of 21 skincare and treatment cream samples, indicating the target audience (men, women, and children) for each product, and potential diseases were analyzed in terms of the hidden risk of microplastics. To determine the exact number of microplastics to which adults and children are exposed over the course of a year, in-depth research was conducted on the cosmetic care and treatment products used by over 354 respondents from Romania. This study used a free, self-reported questionnaire method, which took into account consumer habits and preferences, as well as any potential medical conditions that could affect exposure. Optical microscopy and micro-FTIR revealed a total of 109 microplastics, with different sizes, colors, and shapes (i.e., fragments and fibers) and various chemical compositions, including mixtures of polymeric and natural structures, as well as 100% synthetic materials, e.g., polyethylene and polyester. The potential health risk of exposure to microplastics in certain cosmetic formulations for adults was assessed by calculating various risk indices, such as the polymer risk index (H), pollution load index (PLI), dermal plastic absorption (DPA), chronic daily dermal exposure (CDDE), risk to human health from dermal absorption (RHHDA), and estimated annual dermal absorption (EADA). These indices were calculated based on the medical conditions and application areas indicated on the labels of the analyzed creams (i.e., skincare and treatment), for both adult and children’s categories, using the fingertip unit (FTU) method for estimating the cream amount. The plastic toxicity of the analyzed samples was assessed using the H and PLI indices. The risk of microplastics to human health from dermal exposure was assessed using the DPA, CDDE, RHHDA, and EADA indices, which showed concerning results regarding the presence of these particles in cosmetic formulations.

## 1. Introduction

Skin is the largest organ in the human body, and according to several studies, 60% of used cosmetic products are absorbed directly into the body. Toxins and potential carcinogenic ingredients in these products, including microplastics [1,2,3,4,5,6], reach the body directly, whether through topical application or systemic exposure, without being filtered by the liver or other protective organs, unlike exposure via inhalation or ingestion. Usually, these concerning ingredients should be highlighted on the product labels. However, labels on cosmetics are often difficult to interpret. The cosmetic industry lacks strict regulation, making it hard for consumers to understand these labels, and predictably, it can be difficult to build trust in these products.

The term “organic” can only be used if all the ingredients in the cosmetic product are organic. Usually, the organic label appears on cosmetic products that contain 70% certified organic ingredients. In this case, 30% may be synthetic products that can pose a risk to healthy skin. The cosmetics industry has a “blameless until proven otherwise” approach to ingredients used in products [7,8]. Unless an ingredient used in skincare products has been proven harmful to health, it is considered safe [9,10]. This classification is also supported by the United States Food and Drug Administration (US FDA), and can hardly be considered to be strictly in the interest of consumers [5,6]. A label of “natural”, “safe”, “pure”, “fragrance-free”, “paraben-free”, “microplastics-free”, “dermatologically tested”, or “organic” is often not what it seems, unless it has certifications to demonstrate and support these labels [11]. The last time the FDA evaluated the safety of sunscreen ingredients was in 1978. At that time, the FDA set out to develop quality standards for the safety and efficacy of sunscreen products, but this has not been completed to date, so US manufacturers have no restrictions on the use of certain ingredients in creams whose safety for health has not been proven [11,12].

Certain chemical ingredients in creams, when exposed to solar radiation, can undergo various chemical transformations, i.e., reactions between active and inactive ingredients and the epidermis. Toxic reactions include inflammation, allergic reactions, and effects on cellular DNA [13]. For example, a series of studies have shown that sunscreens contain chemical ingredients that can cause cancer. Basically, this type of cream blocks the absorption of vitamin D produced by sunlight [12,13,14]. Various studies have shown that many chemical ingredients, including microplastics, in skincare and treatment creams have toxic properties that are absorbed by the skin and reach the circulatory system [5,6,15], and these examples can continue infinitely. What can be done in this regard? Obviously, legislation and strict control of cosmetic products [16,17] are necessary, with manufacturers taking responsibility for labels, as well as for advertising, to convince consumers to use these products. Until any of the regulations to be adopted, legislative solutions are necessary to tackle these problems. As previously mentioned, there are many toxic risks related to all sorts of products or types of residues that come into contact with the skin. One of them is a well-known plastic, a widespread synthetic or a semisynthetic polymer.

On the macro-scale, plastic may take many forms and can be used for different commercial purposes, making it easy to be identified on shelves in stores. It is used for shaping brands’ identity or is included in different polymeric compounds as wearable products or wrapping materials. All these aspects complement our day-by-day life and bring comfort on a regular basis, but they also add some major risks for health, mostly given by the presence of very small residual plastic particles either in the air, water, food, cosmetics or skin treatment solutions.

The micro-scale is probably where the unseen environment has the most significant impact on our ecosystem, and is where invisible fragments of plastic, known as microplastics (MPs), which range in size from 0.1 to 5000 µm, pose the greatest risk to human health. Microplastics are particles that meet several conditions, such as the following [5,6]: (i) they are synthetic materials with a high polymer content; (ii) they are solid particles; (iii) they are smaller than 5 mm in size; (iv) they are insoluble in water; and (v) they are not degradable or are very poorly degradable. Despite the availability of sophisticated analytical equipment, the full investigation of the potential of microplastics in topically applied products directly to the skin has met with several limitations and gaps [6]. These challenges cause major issues in experimental design because of the lack of guidance and standardization that could help the transition from qualitative detection to reliable and quantitative assessment of MPs in these products. Furthermore, no risk levels, reference doses, or other maximum allowed values are available at the moment, and the current understanding of microplastic analysis in cosmetics and pharmaceuticals is limited by the trade-off among three key performance factors: sensitivity, analytical yield, and cost. In this respect, it is difficult to optimize a method or an investigation process that could cover all the above-mentioned factors. High-sensitivity techniques capable of detecting and identifying tiny particles, such as FTIR or Raman micro-spectroscopy, are characterized by low throughput (the analysis of small areas or individual particles requires a lot of time) and high costs, mainly due to the costly equipment, the provision of consumables throughout the analysis period (liquid nitrogen) and the long working time for the identification and chemical, structural characterization of each microparticle [6]. In contrast, methods that offer high throughput and low cost, such as optical microscopy, do not have the sensitivity and chemical specificity necessary for reliable identification, especially of small or colorless particles, but can be successfully used in the quantification and primary determination of the shape, color, and sometimes even the size of these microparticles.

Based on well-known global data from various studies [9,12,18,19,20,21,22], reports [4,7,10], guides [16,17], social media influencers (SMIs) [23,24], and media news, the authors’ have conducted extensive research related to the safety of using cosmetics at the consumer level in Romania [25], particularly related to skincare and treatment cream products. In this regard, in this study, several points were taken into consideration in choosing the cream types: (i) the preference of customers related to a brand image in terms of attractiveness, trustworthiness, and marketing; (ii) age category; (iii) gender; (iv) label composition of skincare/treatment creams; (v) concern related to natural vs. organic or organic vs. lab-created ingredient formulations; (vi) consumer well-being; (vii) customer habits; (viii) exposure pathways in terms of used cream category (i.e., skincare or treatment).

Considering the widespread presence of microplastics in the environment and the potential health risks linked to exposure, one of the main objectives of this research is to highlight the hidden dangers associated with microplastics found in cosmetic skin care and treatment products. This analysis is based on preliminary studies conducted by the authors, which involved isolating microplastics from various cosmetic products. These products ranged from simpler items, such as micellar waters, to more complex ones like skincare and treatment creams. The previous studies [5,6] included morphological and chemical characterization of the microplastics, as well as the potential sorption mechanisms of other emerging contaminants on their surfaces. The present research continues, and at the same time extends, the investigations to skincare and treatment products with direct reference to the hidden risk of microplastics due to exposure through dermal contact. It is important to note that this risk has not yet been clearly quantified in the literature, and no correlation has been established so far. Therefore, another objective of this study lies in the novel correlations taken into consideration for the first time between several key indices, i.e., polymer risk index (H), chronic daily dermal exposure (CDDE), dermal plastic absorption (DPA), and the risk to human health caused by dermal absorption (RHHDA), which were calculated.

## 2. Materials and Methods

### 2.1. Materials and Reagents

All reagents used in this research were of high purity (HPLC grade), and mainly purchased from Merck KGaA, Darmstadt, Germany, Carl Roth, Karlsruhe, Germany, and Biosolve Chimie SARL, France. They were the following: ethyl alcohol, C_2_H_5_OH p.a., min. 99% (*v*/*v*), M = 46.07 g/mol; toluene, C_6_H_5_CH_3_, M = 92.14 g/mol; acetone, C_3_H_6_O, 58.08 g/mol; nitric acid 65%; HNO_3_; and sodium hydroxide, NaOH, p.a., min. 99.02%. Deionized water (with a conductivity of less than 0.5 µS/cm at 25 °C) was used in both the sample preparation processes and for the sterilization of materials and utensils used in the preceding stages.

Products from the skincare and treatment cream category were randomly selected from stores (i.e., cosmetics and pharmaceuticals), taking into account customer preferences, brand, cost, and best-selling products, as determined by a previous survey conducted by the authors [25]. On the other hand, another survey was conducted to examine consumers’ knowledge about the impact and risks of microplastics on both the environment and humans. The survey used a questionnaire divided into two sections. The first section aimed to identify respondents’ preferences for skincare and treatment creams, while the second focused on their understanding of the term “microplastics” and its effects on the human body. The survey was completed on the Google Forms platform by 354 respondents from Romania, including both urban (237 respondents) and rural (117 respondents) areas. A total of 274 women (77.40%) and 80 men (22.60%) participated in the study. Respondents ranged in age from 18 to 20 years (16.38% of the total) to over 60 years (5.65%), with the largest age group being 30–39 years (23.73%). Overall, 266 respondents (75.14%) used both skincare and treatment creams, while 51 (14.41%) primarily used treatment creams, and 37 (10.45%) mainly used beauty or skincare creams, as indicated in Table S1. The term “microplastic” was well known to 132 respondents and very well-known to 27 respondents, while 34 and 58 respondents, respectively, had little or no knowledge of it. A total of 153 respondents (43.22%) were aware that skincare and treatment creams may contain microplastics, and 309 respondents (87.29%) expressed significant concern about their presence in products. Several respondents either totally agreed (47 respondents, 13.28%) or agreed (143 respondents, 40.40%) that they would purchase more expensive products—such as skincare and/or treatment creams—that do not contain microplastics, while 101 respondents (28.53%) were undecided. Respondents to this questionnaire agreed that microplastics are highly toxic because MPs can contain and accumulate hazardous substances (131 respondents), are transported along the food chain (138 respondents), and can absorb and transport other emerging contaminants (139 respondents). Environmental pollution with microplastics, especially microbeads from personal care and cosmetic products, represents a major environmental and health problem, because their small size facilitates ingestion by living organisms, transfer along the food chain, and transport of toxic chemicals, including endocrine disruptors, with potential negative effects on ecosystems and human health, according to the study conducted by Guerranti et al. [26]. Starting from the main idea previously presented, 166 respondents (46.90%) agreed with the idea that the cosmetics and personal care products industry is a generator of microplastics, while 80 respondents (22.60%) completely agreed.

The selected samples included ten skincare creams and eleven treatment creams (Table S2), indicating the target audience (men, women, and children) for each product and potential diseases. The desire to look good and the pursuit of a long, healthy life, by increasing education in cosmetology, have significantly contributed to the growth of the skincare cosmetics market. When the term “natural” is associated with these products, sales tend to increase dramatically, reflecting a strong consumer trust. Natural moisturizing face or body creams for all skin types are innovative products used by both women and men, regardless of age, with beneficial potential for the epidermal tissue. Some of these products are recommended for skin regeneration, improving the tonicity of the epithelial tissue, and nourishing and hydrating in depth, with emphasis on their vitamin and antioxidant content [27,28,29,30,31]. Furthermore, many products advertise the healing power of plants, which help develop natural collagen crucial for regeneration and rejuvenation. Usually, creams for adults contain more active ingredients designed to target various skin concerns that are not relevant to children’s skin. Healthy baby skin does not need intensive nourishment, whitening, or anti-aging effects. Consequently, baby creams are typically simple in formulation, keeping in mind that children’s skin has a higher pH level than adults’ (pH 7 for children and pH 5 for adults) and produces less sebum. As a result, baby creams usually contain either mineral oil or lanolin. The main goal of baby cream is protection; it forms an occlusive barrier, a protective film on the skin, which children need.

The cream for the treatment of skin illnesses was chosen depending on the diagnosis, covering a wide range of problems, from fungal to bacterial [13,14,32,33,34,35]. Dermatological issues (e.g., seborrheic dermatitis, psoriasis, zoster zone, eczema, dermatophytosis, rosacea, scabies, etc.) can occur at any age and regardless of gender (Table S1). If left untreated, they worsen, and can even threaten the patient’s emotional balance, causing a decrease in self-esteem [36,37,38].

### 2.2. Microplastic Isolation Method

Microplastics were isolated from the cream sample by treating 1 g of the sample with 3 mL of a 1:1 (*v*/*v*) toluene/acetone mixture. After a 30 min resting period, the sample was ultrasonicated at 40 °C for 30 min. Sample filtration was performed, after the temperature reached 60 °C in a water bath (Precisterm, J.P. Selectra S.A., Barcelona, Spain), using VWR^®^ Grade 413 filter paper (12–15 µm porosity; VWR International LLC, Radnor, PA, USA), and samples were stored in sterilized Petri dishes. Ultrasonication of the samples was performed in an ultrasonic bath, type VWR^®^ Ultrasonic Cleaner USC—TH (VWR International LLC., Radnor, PA, USA), and filtration was carried out with a vacuum stainless steel filter Manifold, with 3 work stations (Labbox Labware, Barcelona, Spain) connected to a Chemker 300 PTFE vacuum pump with a flow rate of 18 L/min (Restek Corporation, Bellefonte, PA, USA). The isolation procedure was achieved in triplicate for each cream sample.

### 2.3. Analytical Techniques

#### 2.3.1. Optical Microscopy

Microparticles isolated on filters were investigated by optical microscopy (OM). Magnification levels used to examine specific features of microparticles ranged from 6.5× to 100×, depending on the microparticles’ complexity and size. Optical microscopy was performed using Primo Star and Stemi 2000-C microscopes (Carl Zeiss Microscopy GmbH, Jena, Germany), which are valuable tools for examining and characterizing the samples under both transmitted and reflected light. The images were captured using ZEN 2012 (Version 1.1.2.0) and a Axiocam 105 digital video camera (Carl Zeiss Microscopy GmbH, Jena, Germany).

#### 2.3.2. Micro-Fourier Transform Infrared Spectroscopy

Micro-Fourier Transform Infrared Spectroscopy (micro-FTIR) is one of the most frequently utilized analytical techniques for chemical and morphological characterization of microplastics in terms of chemical bonding and molecular structure. This nondestructive and noninvasive method is used to analyze small chemical differences in samples, including chemical area and line mapping, and FTIR imaging, through particle analysis and multi-spot techniques testing. Micro-FTIR analysis was performed using a Vertex 80v spectrometer, equipped with a diamond ATR crystal accessory and a Hyperion 2000 microscope (Bruker Optics GmbH & Co. KG, Ettlingen, Germany). The Hyperion microscope, which covers the spectral range of 600–7500 cm^−1^, has a spectral resolution of 0.2 cm^−1^ and an accuracy of ±1 µm, enabling both visual examination and infrared analysis of samples. The chemical structure of the polymer was identified using OPUS v.7.5 software, based on the internal database.

### 2.4. Quality Assurance and Quality Control

The sample preparation and physicochemical analysis steps are crucial in ensuring the accuracy and reliability of the final data. In this regard, it should be noted that strict security measures were adopted throughout the research to avoid sample contamination, in accordance with the protocols proposed by several authors [39,40,41] inside the cleanroom (ISO 14644-1:2015, Class 1000-ISO6) [42]. The analyzed creams were unsealed directly in the cleanroom, without involving tubes, Teflon containers, or other utensils necessary for handling the samples outside the laboratory. All liquid reagents were filtered before usage through filter paper with 12–15 µm porosity, VWR^®^ Grade 413 (VWR International, Radnor, PA, USA), and all laboratory activities were conducted on surfaces previously cleaned with 70% ethanol, following the procedure described by Zhang et al. (2021) [43].

In terms of the user safety measures: (i) non-synthetic clothing and cotton lab coats were worn to prevent textile fibers from detaching; (ii) sterile disposable caps, gloves and masks were used, as well as sterile disposable equipment when necessary. Furthermore, the glassware were thoroughly cleaned by washing with distilled water and 2% nitric acid, then sterilized at 100 °C for 48 h in a Venticell^®^ forced convection oven (BMT Medical Technology Ltd., Brno, Czech Republic), and were covered with aluminum foil when not in immediate use. The filtered ethanol and the ultrapure water were used to rinse the glass jars at least three times before usage.

The equipment used for this study, including 5-place heating shaker, oven, water bath, ultrasonic bath, filtering system, and vacuum pump, were covered with aluminum foil when not in use. In the same manner, the filters were transported safely to the spectroscopy and optical microscopy laboratories for analysis. Taking this approach to ensure sample security during the preparation, the testing and analysis stages are extremely important in minimizing sample contamination.

It is important to note that the filters used for reagents and blank solutions (the sample quantity was replaced with distilled water) were examined after filtration under microscopy (i.e., both techniques), and the results revealed no traces of microplastics on their surface. The isolation method and analyses of microplastics were achieved in triplicate.

### 2.5. Data Analysis

The polymer risk index (H) is an ecological risk assessment tool for microplastics, calculated according to Equation (1), by combining the abundance of different types of polymers (e.g., polyethylene, PE; polypropylene, PP; polyvinyl chloride, PVC; and polystyrene, PS) in an environment with their chemical hazard scores, to identify the potential risk to ecosystems and human health, indicating that polymers such as polyvinyl chloride (PVC), polyacrylonitrile (PAN) or polyacrylamide (PAA) can be much more dangerous than common polymers, even if they are present in small quantities [44]. Therefore, the polymer risk index of MPs was calculated based on Equation (1) [45]:

(1) $$H=P_{i}\cdot S_{i}$$

where *P_i_* represents the percentage of a certain type of polymer out of the total microplastics in an analyzed sample [45], and *S_i_* is the risk factor of polymers according to data from Table 1 established by Lithner et al. [46].

The second parameter used to assess the toxic risk of microplastics in the analyzed cream samples is the concentration factor (*CF_i_*), which is calculated using Equation (2) [45]. *CF_i_* is used in Equation (3) to calculate the pollution load index (PLI) of MPs [47].

(2) $${CF}_{i}=\frac{C_{i}}{C_{0}}$$

(3) $$PLI=\sqrt{{CF}_{i}}$$

where *C_i_* is the concentration of MPs in a specific sample (n∙kg^−1^); *C*_0_ is the minimum average concentration of MPs (n·m^−3^); “n” must be read as particle(s).

Dermal plastic absorption (DPA) refers to the process by which microplastics and their toxic additives enter the body through the skin via topical application, a significant route of exposure, alongside inhalation and ingestion. To calculate DPA, Equation (4) was used according to the research conducted by Banica et al. [44]:

(4) $$DPA=A_{ap}\cdot C_{i}[n\cdot {day}^{-1}]$$

where *A_ap_* is the amount of cream used per application (g·day^−1^); the values for *A_ap_* were established by Long and Finaly [48], as shown in Table 2 (for skincare creams) and Table 3 (for treatment creams); *C_i_* is the concentration of microplastics in the sample (n·kg^−1^).

To estimate chronic daily dermal exposure (CDDE), in the absence of specific formulas, the authors adapted formulas that establish the daily chronic dose of exposure to heavy metals induced by contaminated soils, according to Equation (5) [49]:

(5) $$CDDE=\frac{C_{i}\cdot SA\cdot ED\cdot EF}{BW\cdot AT}\cdot CF[n\cdot {kg}^{-1}\cdot {day}^{-1}]$$

where *SA* is the exposed skin surface area (cm^2^) [50], *ED* is the exposure time (in this case, for adults, *ED* = 20 years), *EF* is the exposure frequency (day·year^−1^), *BW* is the body mass (in this case, for adults, *BW* = 70 kg), *AT* is the average exposure time (days), and *CF* is the conversion factor (*CF* = 1 × 10^−6^) [5].

The risk to human health caused by dermal absorption (RHHDA) of MPs in creams was calculated according to Equation (6), taking into account the polymer risk index (H) calculated with Equation (1).

(6) $$RHHDA=NA\cdot A_{ap}\cdot H$$

where *NA* is the number of applications (NA = one application·day^−1^).

The estimated annual dermal absorption (EADA) of MPs by the human body following the use of potentially contaminated creams was calculated using Equation (7):

(7) $$EADA=NA\cdot A_{ap}\cdot C_{i}[n\cdot {year}^{-1}]$$

## 3. Results and Discussion

### 3.1. Optical Microscopy

The first method used to detect microplastics in skin cream samples was optical microscopy. Although this is a primary analytical investigation technique, it does not provide complete information on the morphology and composition of the polymer(s) in the microparticle composition. While not overly complicated, optical microscopy provides important details about microplastics, including their color, morphology, shape, size distribution, and quantity. However, this method cannot determine whether the microplastics are synthetic, natural, or a mixture of both. The two most significant aspects highlighted through optical microscopy are the size distribution of microplastics in a specific sample and their shape. This technique enables the visual quantification of microparticles, which is essential prior to any further investigation. Optical microscopy revealed the isolated microparticles on the filter’s surface. The color and shape undertaken characterization enabled the quantification of the total number of MPs (see Table 4 and Table 5 and Figures S1 and S2). It is important to note that the investigation also covered control filters and filters through which the risk of reagent contamination was verified; the result in the latter case was zero microparticles (zero contamination degree).

According to the data presented in Table 4 and Table 5, the quantification and classification of microparticles were based on the color (i.e., black, blue, red, purple, gray, brown, green, and yellow) of the identified microparticles. Regarding the investigated skincare creams, most microparticles were identified in the C_1_ sample, and at the opposite pole, the fewest microparticles were found in the C_3_ and C_5_ samples (Table 4). The colors of microparticles were, in descending order, blue (21), black (11), brown (6), gray (5), red (4), yellow (2), and purple (2). Sample C_6_ presents a variety of microparticles, related to colors, which means seven microparticles. The blue color prevailed, being identified in all nine of the skincare cream samples (Table 4). Black microparticles were identified in six samples (C_1_, C_2_, C_3_, C_4_, C_6_, and C_7_), while gray and brown microparticles were identified in five samples (C_1_, C_3_, C_6_, C_8_, and C_10_) and four samples (C_3_, C_4_, C_5_, and C_6_), respectively. Purple and yellow microparticles were detected in two samples (Table 4), while red microparticles (3) were found in samples C_1_, C_2_, and C_6_.

Taking into consideration the treatment cream samples, the obtained data (Table 5) were unexpected in terms of quantification and colors. Thus, most microparticles were found in sample TC_1_, i.e., 54, with a prevalence of colors, in descending order, from black (22), gray (13), red (7), blue (5), yellow (3), and green and brown (2 each). At the opposite pole, summing the smallest number of microparticles, i.e., ten, were samples TC_4_, TC_5_, and TC_9_. Following the data presented in Table 5, it can be observed that black microparticles had the highest prevalence (111) in the analyzed samples, compared to purple ones (1), which were found only in sample TC_5_. Sample TC_1_ presented the highest number of microparticles, but also the greatest diversity of colors, followed by sample TC_8_ (Table 5). Samples TC_6_ presented three color varieties i.e., black (14), yellow (4) and green (1), while in samples TC_9_ and TC_10_, three colors were also detected, but were different, i.e., black (6), gray (3) and yellow (1), and black (8), blue (4) and red (1), respectively. Particular attention should be given to samples TC_1_, TC_6_, and TC_9_ (Table S1), which are considered natural products and intended for treating children over six months old.

Through optical microscopy, the number of microparticles in both categories of creams was quantified, mainly taking into account their color. Information regarding their size and shape was not fully certified, being assumed only the conclusion that the microparticles presented with a compact, opaque or translucent, whose size and shape varied from micrometric dots to twisted micro-/millimeter fibers (Figures S1 and S2). To the authors’ knowledge, no in-depth studies have yet been conducted on skin care and treatment products classified according to age, which has led to a lack of relevant comparisons with other studies.

A thorough analysis was conducted on the large number of microparticles found in sample TC_1_ (Table 5) using micro-FTIR. This analysis focused on examining the chemical and morphological structures of the microparticles, with particular attention to identifying polymeric structures. The goal was to highlight potential microplastics that may pose risks to children’s health.

### 3.2. Micro-Fourier Transform Infrared Spectroscopy

Spectroscopic methods are essential for identifying the chemical structure of polymers, while microscopic techniques help quantify and clarify the morphological structure of microplastics. Since no single analytical method is sufficient for complete characterization, a multi-method approach is often necessary. Typically, the analytical techniques used for analyzing microplastics are non-invasive and non-destructive. These include optical microscopy, micro-Fourier transform infrared spectroscopy (micro-FTIR), and micro-Raman spectroscopy (micro-Raman).

**Figure 1:**
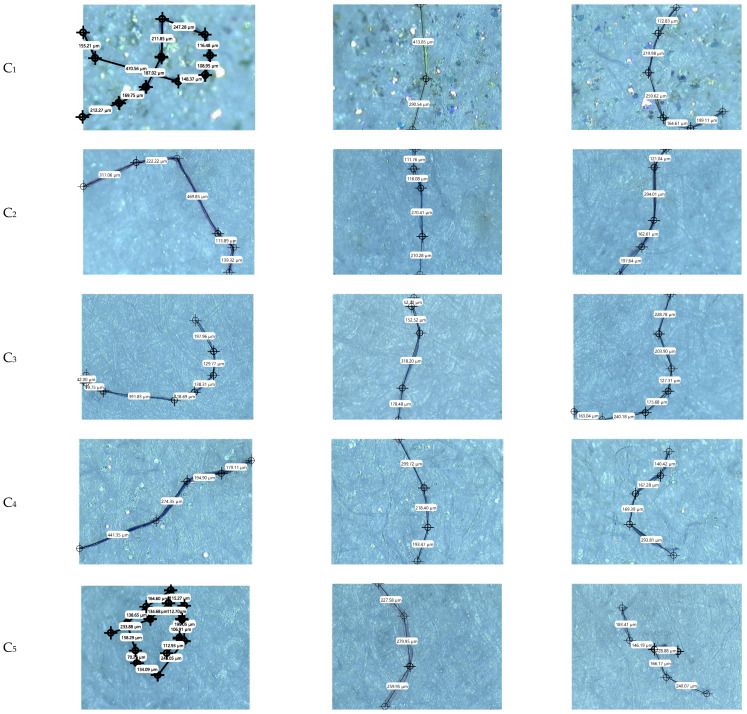

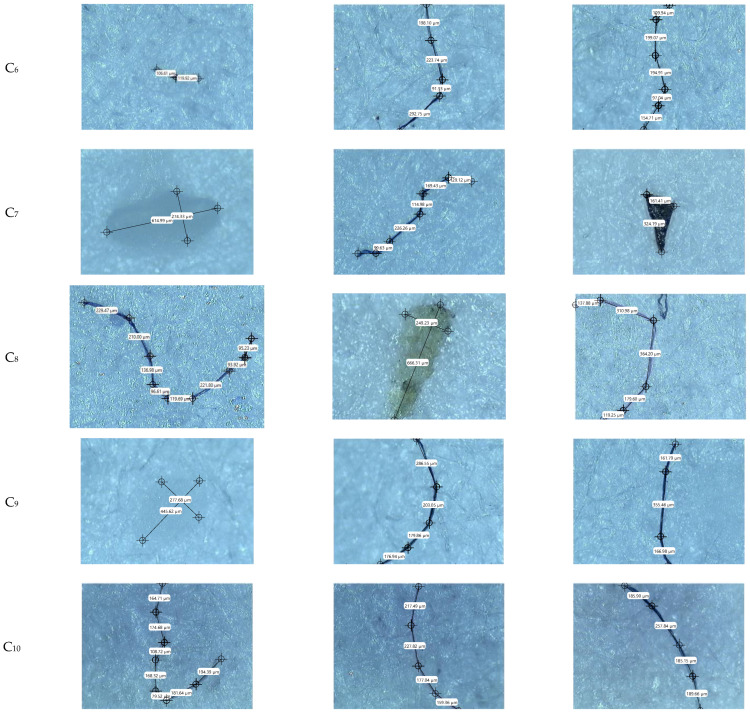
Examples of MP morphologies in skincare samples (in micro-FTIR), mainly fragments and fibers. The fragments ranged in size from 70.91 × 112.11 µm to 1059.26 × 247.80 µm, and the fibers ranged from 226.53 µm to 1797.12 µm in length; micro-FTIR analysis, in terms of size, was performed at three points (complementary data are shown in Table S2).

**Figure 2:**
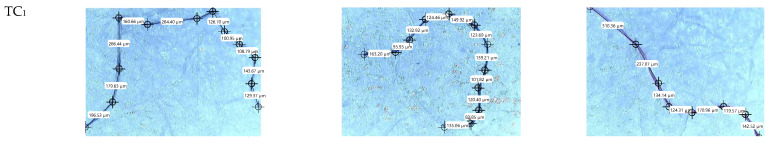

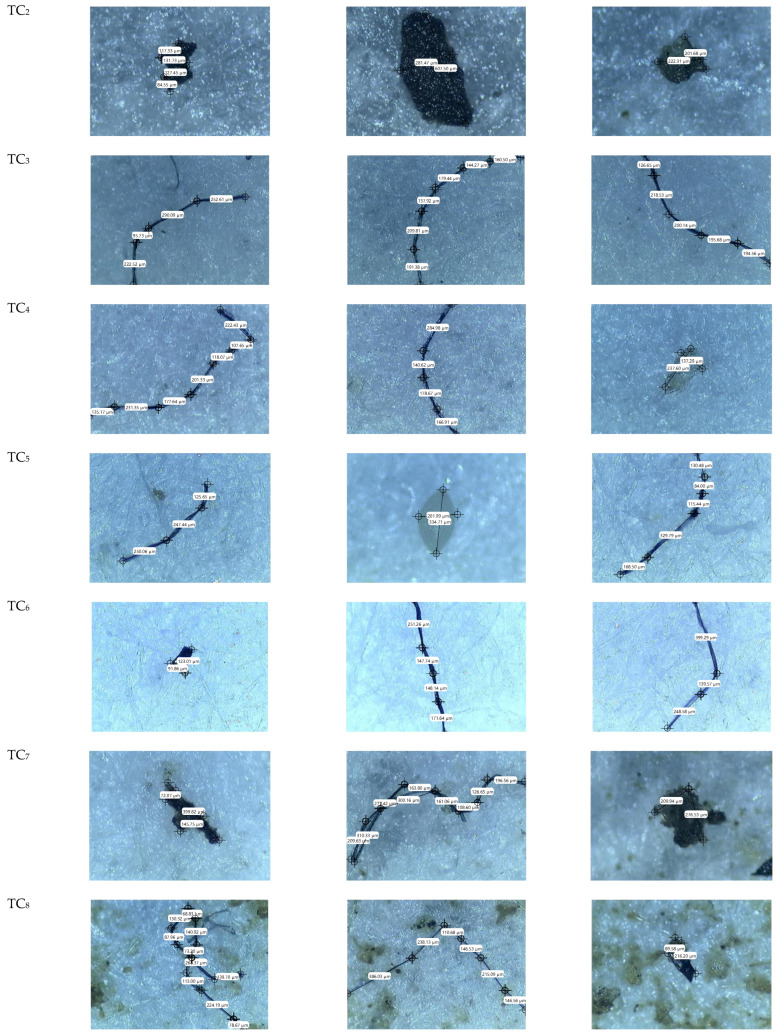

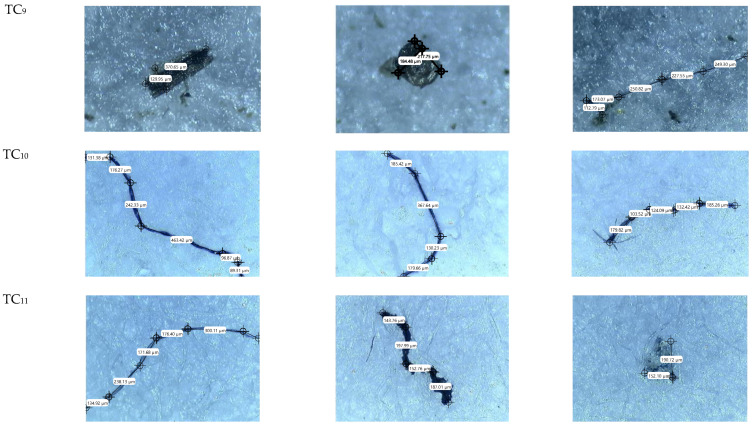
Examples of MP morphologies in treatment cream samples (in micro-FTIR), mainly fragments and fibers. The fragments ranged in size from 99.05 × 68.48 µm to 1304.64 × 145.47 µm, while the fibers ranged in length from 393.61 µm to >1835.88 µm; micro-FTIR analysis, in terms of size, was performed at three points (complementary data are shown in Table S2).

**Figure 3:**
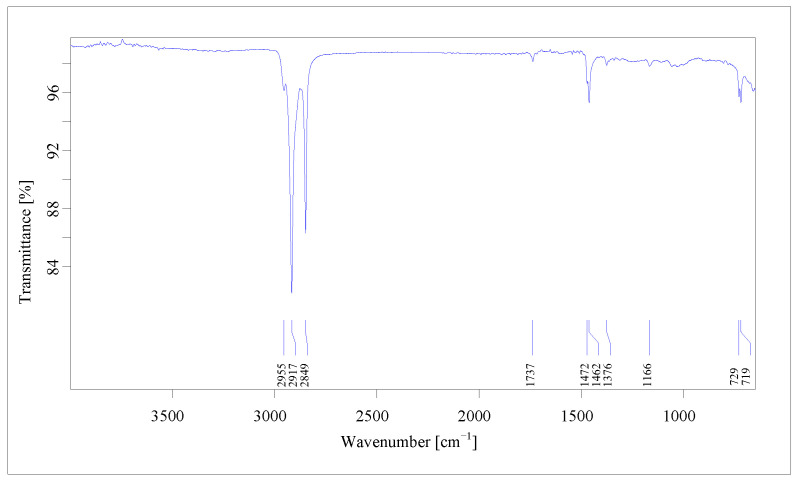
Representative FTIR spectrum for polyethylene identified in treatment cream samples (TC_6.3_, TC_7.4_, TC_9.1_, TC_9.2_, and TC_9.9_, according to Table S4).

**Figure 4:**
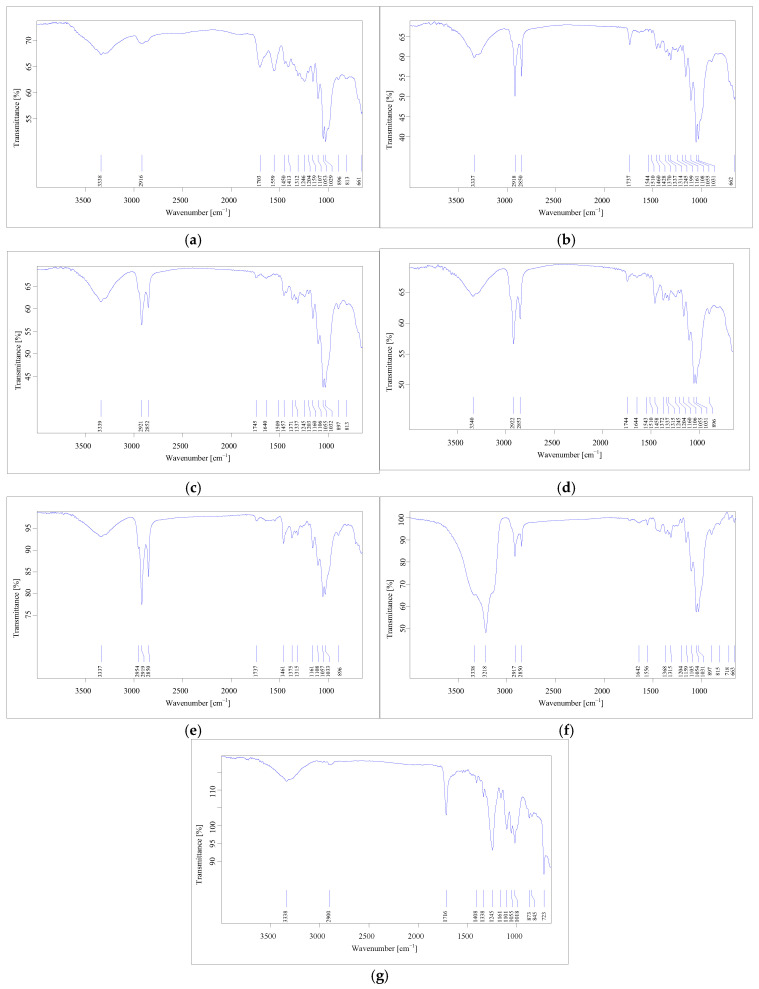
FTIR spectra for microplastic fiber mixtures from treatment cream samples: (**a**) TC_1.1_ = 60% cotton + 40% polyester; (**b**) TC_3.7_ = 60% cellulose + 40% PMMA; (**c**) TC_4.3_ = 70% cotton + 13% cellulose + 11% nylon + 6% PMMA; (**d**) TC_7.1_ = 92% cellulose + 8% polyurethane; (**e**) TC_9.7_ = 50% cellulose + 50% PMMA; (**f**) TC_9.8_ = 65% cotton + 25% nylon + 10% cellulose; (**g**) TC_10.1_ = 50% cotton + 50% PES. The TCx.y represents the code of microplastic identified in the analyzed samples according to Table S4.

Micro-FTIR imaging provided a comprehensive characterization of MPs in terms of chemical structure and morphological properties (Tables S3 and S4, Figure 1 and Figure 2), with data that is reliable and reproducible. Differences in the micro-FTIR spectra of MPs isolated from cream samples were noted, with a focus on the spectral regions and peaks highlighted in Figure 3 and Figure 4. Micro-FTIR nondestructive analysis was used to identify and differentiate natural microparticles from those with polymeric structures, enabling accurate identification even of natural–synthetic mixtures, including polymer-nanocoated natural fibers [51,52]. In this regard, the obtained data highlighted that in samples TC_5_ and TC_11,_ no polymeric structures were identified, with the microparticles being attributed to chemical structures originating from natural materials (cotton, cellulose, resin, and linen). It can be concluded that the two treatment creams (Table S1) are practically “microplastic-free”. In addition, the chemical composition of microparticles was emphasized through the interpretation of FTIR spectra, which considered the weak, medium, and strong vibrational frequencies. In spectroscopy, peak intensities (i.e., weak, medium, strong) and wavenumbers help assign functional groups in organic compounds. In Figure 3, the overlaps of FTIR spectra of the fibers isolated from treatment cream samples are displayed.

A total of 109 microplastics were identified and confirmed, including a mixture of polymeric and natural structures, as well as 100% synthetic materials such as polyethylene (PE) (see Tables S3 and S4). This data was obtained using the spectra library of OPUS software version 7.5.

Micro-FTIR mapping of the ten skincare cream samples revealed 36 synthetic fibers and 14 irregularly shaped synthetic fragments, most of which were translucent (Table S3). Most fibers and fragments were identified in sample C_3,_ i.e., eight fibers of different lengths and compositions and four synthetic fragments (three with a composition of 100% polyethylene, PE, and one white fragment measuring 130.21 × 86.58 µm, with a composition of 60% cellulose acetate and 40% polyacrylic fiber (i.e., poly(methyl methacrylate, PMMA), according to the data presented in Table S3). The cream is labeled as using organic ingredients and is intended for adults aged 30–40, recommended as a restructuring night cream for the face and neck. On the other hand, in sample C_6_ (night cream for face and neck, for women and men, Table S2), a single microplastic was identified with a mixed composition of 70% cotton, 13% cellulose acetate, and 17% polyamide (i.e., nylon), in the form of a fiber with a length of 226.53 µm (Figure 1 and Table S3). Two microplastics were identified in skincare creams C_1_ (vegan, recommended for adults) and C_8_ (organic, for men), in the form of fibers, the composition being natural structures with polymetric ones, except for sample C_8.1_, identified as an irregular white fragment, with a composition of 60% polyester (PES) and 40% cellulose acetate (Table S3). Out of a total of fifty microplastics identified and analyzed in terms of chemical and morphological composition, only five samples showed 100% polymer, namely PES, with the samples taking the form of fragments (three microplastics in C_3_) and fibers (two microplastics in C_7_). Optical microscopy and micro-FTIR revealed four microplastics (two 100% polyester fibers and two medium-sized, irregular, translucent fragments with a mixed cotton–polyester composition, Table S3 and Figure S1) in sample C_7_, recommended baby skincare, which represents an issue.

Concerning treatment creams intended for adults but also children, labeled “natural” or “non-organic” (Table S2), a total of 59 microplastics were identified and analyzed morphologically and compositionally by micro-FTIR (Table S4), out of a total of 255 microparticles identified by optical microscopy (Table 4 and Figure S2). Micro-FTIR mapping showed the absence of microplastics in two treatment creams, TC_5_ and TC_11_ (Table S4), intended for adults and children, or only adults, respectively, which are recommended for the treatment of various skin diseases (Table S2), although 10 and 26 microparticles, respectively, were initially identified by optical microscopy (Table 4 and Figure S2). In the treatment cream category, micro-FTIR identified a greater number of irregular fragments than in skincare creams (only 14), more precisely 29 (i.e., six in TC_2_, mostly black in color; one in TC_4_; two in TC_6_; seven in TC_7_; nine in TC_9_; and four in TC_10_, mainly translucent, with sizes ranging from 99.05 × 68.48 µm to 1304.64 × 145.47 µm, Figure 2 and Table S4).

On the other hand, most of the identified fragments had a chemical composition in the form of a mixture (i.e., mainly cellulose acetate–PMMA), but five fragments (TC_6.3_, TC_7.4_, TC_9.1_, TC_9.2_, and TC_9.9_) were clearly identified using OPUS v.7.5 software as being 100% polyethylene, with the FTIR spectrum shown in Figure 3. Most microplastics, ten in number (fibers and fragments), were identified in samples TC_9_ and TC_10_ (Table S4), non-organic treatment products intended for adults and children (TC_9_), recommended for inflamed skin, with a soothing and antibacterial effect.

For a more accurate interpretation of the structure of microplastics identified as mixtures, the chemical structures of polymers were analyzed by evaluating FTIR spectra, with direct reference to functional groups and peak assignments, as well as an analysis of the recorded intensities. The FTIR spectra analysis, which ranges from 3056 to 3026 cm^−1^, shows a sum of the O-H stretching assigned to the hydroxyl group of cellulose structures. The signals observed around 895 cm^−1^ are associated with the stretching and bending vibrations of the CH, CH_2_, and CH_3_ groups. These signals are derived from the structures of poly(methyl methacrylate), nylon, polyurethane, and cellulose acetate, which may or may not include the C-ring (Figure 4).

The peaks resulting from these vibrational modes are present in nearly all of the analyzed cream samples. Furthermore, the bending of C=O groups from amide structures in nylon shows peaks in the ranges of 1245–1278 cm^−1^, 1723–1727 cm^−1^, and 1905–1916 cm^−1^. Weak- to medium-intensity bands were noted in all recorded spectra. From an analytical perspective, the C-H stretching region (2800 to 3056 cm^−1^) is particularly significant, as it displays several distinct absorption differences in the spectra, characterized by strong and medium peaks (Figure 4). Variations in vibrational frequency may arise from various intermolecular and intramolecular interactions, potentially affecting molecular structure, such as steric effects. Several values obtained from the FTIR spectra, expressed as wavenumbers, show the symmetric CH_2_ stretching mode shifted to higher wavenumbers. This shift can lead to an overlap with the symmetric CH_3_ stretching modes, causing the combined CH_3_ and CH_2_ signals to appear as a broad band, as illustrated in Figure 4.

### 3.3. Data Analysis

To evaluate the potential risks to the environment and human health from using skincare and treatment creams contaminated with microplastics, this study aims to assess five quantitative risk indices. The polymer risk index (H) measured the inherent hazard related to the amount of microplastics and the types of polymers found in the samples. To evaluate the level of microplastic contamination in cosmetic and dermatocosmetic samples, CF_i_ and PLI indices were calculated. Additionally, DPA was determined based on the application area, and CDDE by the gender of consumers (i.e., women or men). Collectively, these indices provide comprehensive information for interpreting both the presence of microplastics and their potential environmental and health risks.

The polymer risk index (H) is a valuable tool for evaluating the potential risks associated with using skincare and treatment creams that may be contaminated with microplastics. The H-index was determined for nineteen cream samples, with the results for the two categories (soothing and treatment) summarized in Table 6.

Among these, the lowest H values were observed in samples C_6_ (H = 6676.00) and TC_6_ (H = 35,927.00), while the highest were in C_1_ (H = 226,360.00) and TC_9_ (H = 159,047.00). Notably, no polymers were detected in two treatment creams (TC_5_ and TC_11_). According to Lithner et al. [46], who established five risk thresholds, the cosmetic samples range from high to very high risk, whereas the treatment creams fall within the very-high-risk category, with H values exceeding 10,000.

It is also important to note that five different polymers were identified across both cream categories: PMMA, PU, PES, PE, and PA. These polymers are commonly used in the cosmetics industry because of their functional properties, such as stabilizing formulations and enhancing texture or film formation.

However, the literature highlights that certain polymers, especially persistent and poorly biodegradable ones, can contribute to the accumulation of microplastics in the environment and can generate long-term ecotoxicological effects [53,54,55].

**Figure 5:**
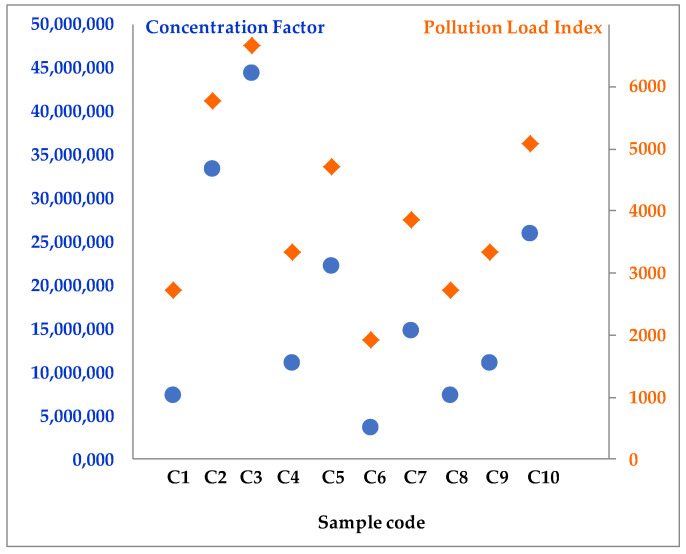
Concentration factor, CF_i_ (blue oval bullet), and pollution load index, PLI (green rhombus bullet) for skincare cream samples.

**Figure 6:**
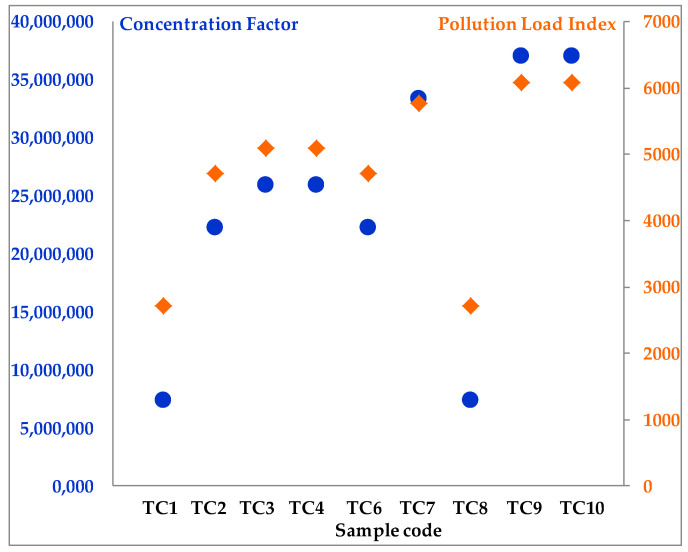
Concentration factor, CF_i_ (orange oval bullet), and pollution load index, PLI (dark-blue rhombus bullet) for treatment cream samples.

To better understand the level of microplastic pollution in the cream samples analyzed (Figure 5 and Figure 6), the pollution load index (PLI) was also calculated, an index closely related to the concentration factor (CF_i_). PLI is widely used in assessing the cumulative level of contamination and allows for the classification of samples according to their potential risk to human health. The calculated H values for the treatment cream samples are presented in Table 6.

The PLI for each sample from the two categories of creams (i.e., skincare and treatment) analyzed contributes significantly to the stability of the potential health risk to users of the two product categories. The high values of the PLI showed a high cumulative load with microplastics, determined by high concentrations of polymers (CF_i_) identified in the analyzed samples.

From a human health risk perspective, these results are particularly relevant, given that both skincare (Figure 5) and treatment creams (Figure 6) are applied directly to the skin, sometimes over large areas and for long periods.

With treatment creams, frequent use (one to three times daily, depending on medical indications) may increase overall exposure to microplastics, thus sustaining the high PLI recorded values. The values obtained for both categories of creams emphasize the importance of carefully assessing the polymer content in cosmetic and dermatocosmetic products. Additionally, there is a need to develop safe formulations for consumers that maintain efficacy without increasing risks to human health.

Depending on the amount applied daily, but also on the area of the body where the skincare cream is applied, DPA was calculated in accordance with studies conducted by Banica et al. [44]. Aristizabal et al. revealed in their research that 4000.00 MPs were identified in face and hand skin creams [2]. In the current research, MP concentrations ranged from 1.00 n·day^−1^ (C_6_) to 44.50 n·day^−1^ (C_1_). It should be noted that very high values of H were recorded in these samples. The DPA values for each skincare cream sample are detailed in Table 7.

Using the same calculation method applied to skincare creams, the DPA was also calculated for treatment creams. The recorded values are presented in Table 8. It is worth noting that no MPs were identified in the TC_5_ and TC_11_ samples.

**Figure 7:**
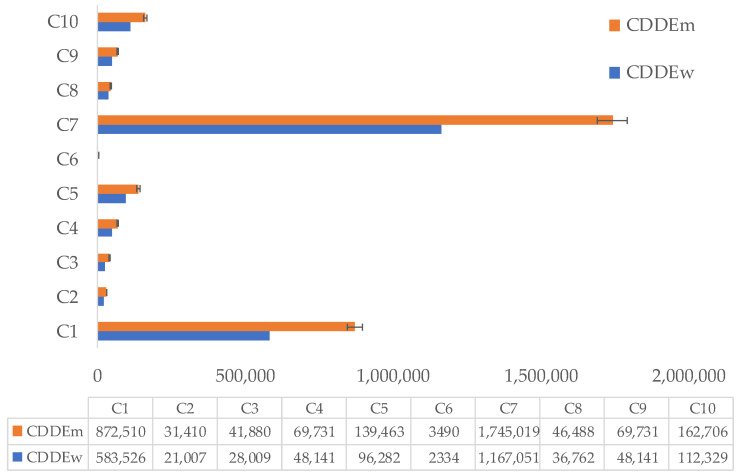
Chronic daily dermal exposure (expressed as n·kg^−1^·day^−1^) for ten skincare cream samples (i.e., C_1_ to C_10_) used by women (blue color) and men (orange color); CDDEw and CDDEm is abbreviation for chronic daily dermal exposure in the case of women and men, respectively.

To estimate chronic daily dermal exposure (CDDE), the authors separately estimated the dermal exposure area for adults and assumed an exposure duration of 20 years. The CDDE values calculated for the ten skincare cream samples used by women are presented in Figure 7, where the highest value was obtained for sample C_7_ (1,167,051.00 n·kg^−1^·day^−1^), and the lowest value (2334.10 n·kg^−1^·day^−1^) was obtained for sample C_6,_ In the case of men, the highest and lowest values for CDDE were obtained for samples C_7_ and C_6_, respectively.

**Figure 8:**
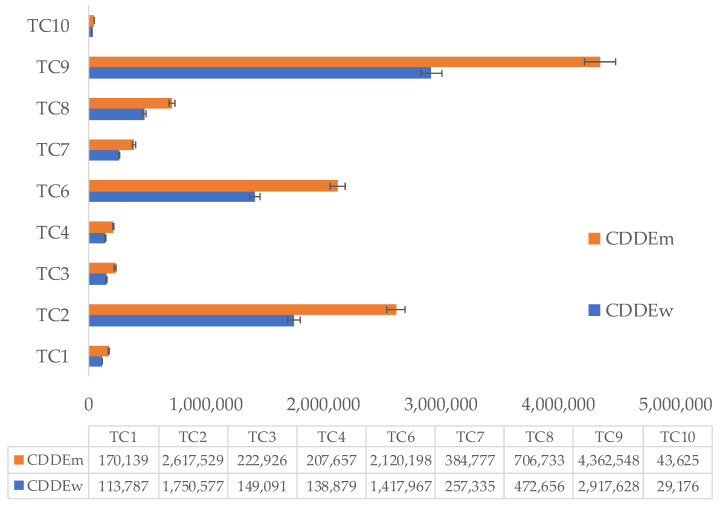
Chronic daily dermal exposure (expressed as n·kg^−1^·day^−1^) for treatment cream samples (i.e., TC_1_ to TC_11_, except TC_5_ and TC_11_—“free microplastics”); used by women (blue color) and men (orange color); CDDEw and CDDEm are abbreviations for chronic daily dermal exposure in the case of women and men, respectively.

For the treatment cream samples, the calculated CDDE values are graphically represented in Figure 8. The highest value was recorded for TC_9_ (2,917,627.50 n·kg^−1^·day^−1^), and the lowest was reported for TC_10_ (29,176.28 n·kg^−1^·day^−1^). As expected, in the case of treatment creams used by men, the highest and lowest CDDE values were recorded in the same range as those used by women, according to Figure 8.

These results highlight significant variability among the analyzed samples, for both skincare and treatment creams, for the adult category (i.e., women and men). Dermal exposure to microplastics depends on the formulation of each product, the concentration of MPs, and the type and physicochemical properties of the polymers present in cosmetic and dermatocosmetic products.

The risk to human health caused by dermal absorption was calculated for both skincare creams (Table S5) and treatment creams (Table S6). The RHHDA was calculated for different areas of the body. Regarding treatment creams, the authors consider that such treatment is applied to specific areas rather than to the whole body, as with skincare creams. Also, it is important to mention that the RHHDA was calculated taking into account the polymer risk index (H), and the results cannot be cumulated to determine the total RHHDA.

The RHHDA was calculated for the two types of creams (skincare and treatment cream) on the microplastics identified in one gram of the sample (C_i_). The risk level of the analyzed creams is worrying, with the exposure pathway being dermal, but cumulating with the other two exposure routes (inhalation and ingestion) to which a person is subjected daily. In such cases, the risk level can be higher than the reported data. The authors were not able to report the data for other studies, but the research carried out by Aristizabal et al. in 2024 [2], with the consent of the subjects who participated in the study, reported that >7000.00 microplastics were identified in dermatological samples intended for the scalp and >4000.00 microplastics were identified in dermatological products intended for the face and hands after a 24 h application period. The authors of the study did not specify the amount of dermatological product and the number of applications in 24 h, and assuming that a quantity of dermatological product was used according to the quantities established by Long and Finlay [48] in their research, i.e., 1.50 g cream for the scalp and 5.00 g cream for the face and hands (face, back and fingers). Therefore, it can be mentioned that according to the PAAS National^®^ Version 1.0 [56], the fingertip unit (FTU) method is one of the most useful in terms of accurately assessing dermatocosmetic cream quantity used for a specific purpose (i.e., skincare or treatment) when the directions for use are ambiguous or unspecified by the producers. In other words, the FTU is equivalent to the amount of cream that covers from the tip of an adult finger to the first crease (i.e., the FTU is approximately 0.5 g, which is sufficient to cover both sides of an adult hand; it can be estimated that for children, one FTU is almost 0.25 g) [2,48].

To calculate the RHHDA for the scalp, hands, and face, the amount of treatment cream (coded TC; see Table S2) used in a single application, on average, was considered. Taking into account the polymer structures of the microplastics identified by micro-FTIR spectroscopy (Table S4) in two different dermatological products (TC_2_, i.e., PMMA, PU, and PA; and TC_9_, i.e., PE, PMMA, PA, and PU), and the fact that approximately 5.00 g of cream was needed for the application areas, RHHDA could be calculated. The RHHDA values for the scalp area were therefore found to be 223,924,800.00 for TC_2_ and 166,999,350.00 for TC_9_. These data are approximately consistent with those obtained by Aristizabal et al. [2] in their study. In this regard, for a better understanding and correlations between the current study and those conducted by Aristizabal et al. [2], the RHHDA was calculated. Thus, RHHDA values recorded a number of 746,416,000.00 microplastics at scalp level and 556,664,500.00 microplastics at the face and hands combined level, respectively.

Comparing the RHHDA values obtained by both studies (i.e., the current study and Aristizabal et al. [2]), it can be concluded that the RHHDA values obtained for TC_9_ sample were higher than those recorded by Aristizabal et al. [2], due to the higher number of microplastics. The results for skincare creams could not be compared with similar data in the literature in terms of the application area and dermatologists’ recommendations for their use on specific or cumulative anatomical parts of the human body. Abbasi and Turner revealed that the highest amounts of microplastics were found in scalp hair after 24 h, followed by the skin of the face and hands [57].

**Figure 9:**
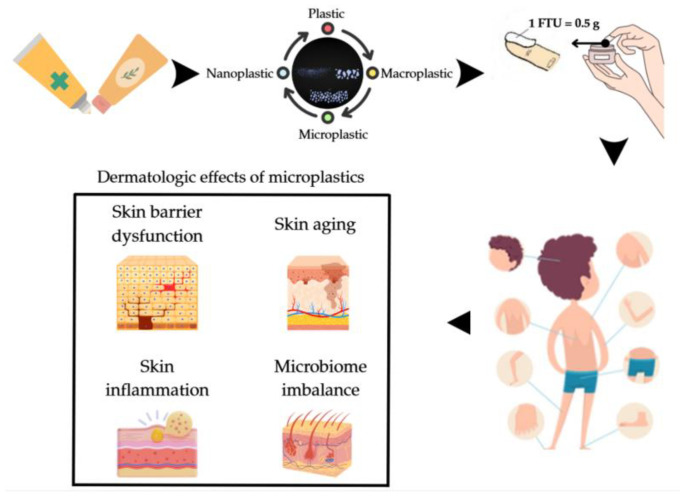
Areas of application of the skincare cream and treatment cream, presentation of the reference dose expressed in FTU, and the dermatological effects of microplastics; FTU is approximately 0.5 g, sufficient to cover both sides of an adult hand; it can be estimated that for children, one FTU is almost 0.25 g [49].

Even though dermal exposure is considered a less dangerous pathway and several factors can influence skin permeability [58], this exposure pathway should not be neglected because the effects on the human body can be very serious. Figure 9 shows the areas of application of creams and the dermatological effects following their application on the human body when contaminated with microplastics.

Among the effects of microplastics on the skin, the following can be noted: (i) skin barrier dysfunction that reduces the stability of the lipid monolayer, weakening the skin barrier [59] and allowing the infiltration of external substances [60]; (ii) microbiome imbalance, caused by disturbing the balance between beneficial and harmful bacteria in the intestinal microbiome [61,62,63,64]; (iii) skin aging that causes oxidative stress and DNA damage [60,65], disrupts cellular metabolism through autophagy and endoplasmic reticulum (RE) stress [65,66], and induces mitochondrial oxidative stress [67]; (iv) skin cancer induced by cell cycle abnormalities [68] and DNA damage [69]; and (v) inflammation that contributes to the development or worsening of inflammatory skin conditions [60,70].

The estimated annual dermal absorption (EADA) for MPs identified in skincare and treatment creams was calculated based on Equation (7), where the number of MPs identified in each cream was taken into account. The amount of cream applied to each area according to Table 2 and Table 3 and the number of days of a year (i.e., 365 days), assuming that the two types of cream are applied daily, and the results obtained are presented in Tables S7 and S8.

According to Table S7, the estimated annual dermal absorption is 32,485.00 n·year^−1^ in cream C_7_ for a daily application of 22.25 g of cream to the entire human body. Considering that skincare creams are most often applied to the face, neck, and hands, the EADA is 1642.50 MPs·year^−1^ for an applied amount of cream of 2.25 g for all three areas. For the face and neck area, the EADA ranges from 1368.75 MPs·year^−1^ to 2737.50 MPs·year^−1^ for an applied amount of cream of 1.25 g.

Treatment creams are used to manage a wide range of skin conditions (from dryness and itching to acne, infections, inflammatory conditions, etc.) [71,72]. The EADA values are presented in Table S8, and the highest amount of MPs released in one year during the use of treatment creams is at the trunk of the body, where the values ranged from 5110.00 n·year^−1^ to 25,550.00 n·year^−1^ for an application of 7.00 g of cream. There are situations where treatment creams are applied to multiple areas of the body to treat a condition, and the EADA increases exponentially depending on the amount of cream used and the frequency of use (e.g., 2–3 times per day). The authors were unable to compare the results obtained for EADA because there are no other studies in the specialized literature that specifically estimate the number of microplastics released onto the human body surface, particularly at the skin level, due to the use of skincare and treatment creams. Instead, two studies, one that estimated the amount of microplastics released into the environment over the course of a year [73] and another that estimated the annual amount of microplastics released from personal care products [74], were used as models for the current research. Thompson et al. [74] revealed that microplastic emissions into the environment range from 10 to 40 million tons per year, and in the best-case scenario, this amount could double by 2040. Sun et al. [75] estimated that 1500.00 tons of microplastics from personal care products are released annually from wastewater treatment plants and enter the environment, while worldwide, the quantity may reach 12,000.00 tons per year.

The results obtained in the present study are worrying, considering that the research focused on only two products from the personal care products category, and the number of cosmetic and personal care products used in a day is much higher. Furthermore, the estimates of Thompson et al. [74] may be realistic, given that there are three main routes of exposure to microplastics (i.e., ingestion, inhalation, and skin/skin contact), and all sectors are now affected by the presence of nano-, micro-, and macroplastics.

## 4. Conclusions

The results of the questionnaire indicate that the use of skincare and treatment creams is widespread among respondents, with the majority using both types of products at the same time, suggesting a high potential for exposure to microplastics through personal care products. The level of knowledge of the term “microplastics” is moderate, with significant differences found between respondents, highlighting the need to increase public awareness of the presence and effects of microplastics in cosmetic products. Although not all respondents are fully aware of the presence of microplastics in cosmetic and treatment creams, the majority express a high degree of concern about their use, reflecting heightened sensitivity to their environmental and health impacts.

A significant proportion of respondents are willing to purchase more expensive cosmetic products, provided that they do not contain microplastics, suggesting significant potential for the development and promotion of alternative, microplastic-free products in the cosmetic market. Respondents perceive microplastics as having a high degree of toxicity, due to their ability to accumulate and transport hazardous contaminants and to transfer along the food chain, a perception that is consistent with the specialized literature. The majority of study participants agree that the cosmetics and personal care industry contributes significantly to microplastic pollution, thus confirming the relevance of this industry as a significant source of microplastics in the environment.

The isolation of microplastics from cosmetic and dermatocosmetic creams was challenging due to their low concentrations, varied physicochemical properties, and interference with the complex organic composition of these creams.

To determine the exact number of microplastics that adults and children are exposed to over the course of a year, it is essential to conduct thorough research on routine products in the cosmetic and pharmaceutical fields. This research should consider the habits and preferences of consumers, as well as any potential medical conditions that may affect exposure. Although the origin of the microplastics found in skincare and treatment creams was not the primary focus of this study, their presence in both organic and vegan samples, as well as natural products, suggests that contamination likely stems from the external environment and the method of application. Additionally, contamination during processing and the materials used in product packaging cannot be ruled out.

In this respect, the high and very high H-index values obtained for all analyzed samples suggest the need for a more stringent evaluation of the use of polymers in cosmetic products, as well as the implementation of strategies that promote safer and more sustainable alternatives. The results highlight the importance of continuously monitoring cosmetic compositions and assessing associated risks. This contributes to substantiating regulatory decisions and promoting responsible practices in the cosmetic industry.

Furthermore, the consistency between CDDE trends and other risk indicators (H-index, PLI, DPA, RHHDA, and EADA) applied in this study strengthens the robustness of the overall risk assessment. This suggests that products identified as high-risk by one metric tend to exhibit elevated values across multiple assessment approaches. This convergence highlights the importance of conducting comprehensive, multi-indicator evaluations when assessing the potential health implications of microplastic-containing cosmetic products.

## Figures and Tables

**Table 1:** Risk factors of polymers and hazard level for several polymers [46].

Polymer Abbreviation	S_i_	Hazard Level
PMMA	1021	IV (1000 < H < 10,000)
PU	13,844	V (H > 10,000)
PES	1177	IV (1000 < H < 10,000)
PE	11	II (10 < H < 100)
PA	50	II (10 < H < 100)

PES—polyester; PE—polyethylene; PU—polyurethane; PA—nylon; PMMA—poly(methyl methacrylate).

**Table 2:** The amount of skincare creams required for applying to different anatomical areas [48].

Body Area	A_ap_ [g·day^−1^]
Whole body	22.25
Face, neck, and hands	2.25
Face	1.00
Face and neck	1.25
Hands	1.00

Note: One application per day was taken into account.

**Table 3:** The amount of cream required for applying treatment creams to different anatomical areas [48].

Body Area	A_ap_ [g·day^−1^]
Scalp	1.50
Face and neck	1.25
Hand (front, back, and fingers)	0.50
Arm	1.50
Elbows	0.50
Foot (top, sole, and toes)	0.75
Knees	0.50
Entire leg	4.00
Trunk	7.00
Entire arm and hand	2.00
Buttocks	2.50

Note: One application per day was taken into account.

**Table 4:** Number of microparticles identified by optical microscopy in 1 g of skincare cream and the content of microplastics determined by micro-FTIR (expressed as n·g^−1^).

Sample Code	Color and Number of Microparticles							Total Microparticles [n·g^−1^]	Total Microplastics (*C_i_*) [n·g^−1^]	Standard Deviation (SD)
	Black	Blue	Red	Purple	Gray	Brown	Yellow			
C_1_	2	6	1	nd	1	nd	nd	10	2	0.58
C_2_	5	4	1	nd	nd	nd	nd	10	9	0.00
C_3_	10	nd	nd	nd	1	1	nd	12	12	0.58
C_4_	1	2	nd	nd	nd	3	nd	6	3	0.00
C_5_	nd	5	nd	nd	nd	1	nd	6	6	0.00
C_6_	1	1	2	1	1	1	nd	7	1	0.00
C_7_	2	3	nd	nd	nd	nd	nd	5	4	0.00
C_8_	nd	3	nd	nd	1	nd	1	5	2	0.58
C_9_	nd	4	nd	nd	nd	nd	nd	4	3	0.00
C_10_	nd	5	nd	1	1	nd	1	8	7	0.00

nd—unidentified; *C_i_*—microplastics per gram of product [n·g^−1^] represents the average value (rounded to an integer value).

**Table 5:** Number of microparticles identified by optical microscopy in 1 g of treatment cream and the content of microplastics determined by micro-FTIR (expressed as n·g^−1^).

Sample Code	Color and Number of Microparticles								Total Microparticles [n·g^−1^]	Total Microplastics (*C_i_*) [n·g^−1^]	Standard Deviation (SD)
	Black	Blue	Red	Purple	Gray	Brown	Yellow	Green			
TC_1_	22	5	7	nd	13	2	3	2	54	2	0.00
TC_2_	12	2	1	nd	3	1	1	nd	20	6	0.58
TC_3_	6	6	nd	nd	9	1	nd	nd	22	7	0.00
TC_4_	3	2	nd	nd	3	2	nd	nd	10	7	0.58
TC_5_	8	nd	nd	1	nd	1	nd	nd	10	0	0.00
TC_6_	14	nd	nd	nd	nd	nd	4	1	19	6	0.58
TC_7_	3	5	1	nd	12	nd	nd	1	23	9	0.00
TC_8_	12	8	5	nd	20	nd	nd	3	48	2	0.00
TC_9_	6	nd	nd	nd	3	nd	nd	1	10	10	0.00
TC_10_	8	4	1	nd	nd	nd	nd	nd	13	10	0.58
TC_11_	17	1	1	nd	6	1	nd	nd	26	0	0.00

nd—unidentified; *C_i_*—microplastics per gram of product [n·g^−1^] represents the average value (rounded to an integer value).

**Table 6:** Polymer risk index (H) values for skincare and treatment cream samples.

Skincare Creams				Treatment Creams			
Sample Code	Type of Identified Polymer	Hi	H	Sample Code	Type of Identified Polymer	Hi	H
C_1_	PMMA	40,840.00	226,360.00	TC_1_	PMMA	40,840.00	40,840.00
	PU	138,440.00		TC_2_	PMMA	37,981.20	149,283.20
	PES	47,080.00			PU	110,752.00	
C_2_	PMMA	40,840.00	221,912.00		PA	550.00	
	PU	179,972.00		TC_3_	PMMA	45,361.57	45,361.57
	PE	1100.00		TC_4_	PMMA	33,522.83	47,019.83
C_3_	PMMA	3063.00	33,117.20		PES	12,947.00	
	PE	1100.00			PA	550.00	
	PES	28,954.20		TC_6_	PMMA	32,672.00	35,297.00
C_4_	PMMA	33,182.50	70,846.50		PA	1525.00	
	PES	37,664.00			PE	1100.00	
C_5_	PMMA	16,336.00	195,344.00	TC_7_	PMMA	42,065.20	44,065.20
	PA	550.00			PA	900.00	
	PU	138,440.00			PE	1100.00	
	PES	40,018.00		TC_8_	PMMA	51,050.00	51,950.00
C_6_	PMMA	6126.00	6676.00		PE	900.00	
	PA	550.00		TC_9_	PE	1100.00	159,047.00
C_7_	PES	36,487.00	37,587.00		PMMA	45,945.00	
	PE	1100.00			PA	1250.00	
C_8_	PES	27,659.50	27,659.50		PU	110,752.00	
C_9_	PMMA	22,802.33	23,352.33	TC_10_	PMMA	37,777.00	38,877.00
	PA	550.00			PE	1100.00	
C_10_	PMMA	40,402.43	40,402.43				

**Table 7:** Dermal plastic absorption values for skincare cream samples.

Body Area	Dermal Plastic Absorption [n·day^–1^]									
	C_1_	C_2_	C_3_	C_4_	C_5_	C_6_	C_7_	C_8_	C_9_	C_10_
Whole body (A_ap_ = 22.25)	44.50	-	-	-	-	-	89.00	-	-	-
Face, neck, and hand (A_ap_ = 2.25)	-	-	-	-	-	-	-	4.50	-	-
Face (A_ap_ = 1.00)	-	-	-	3.00	-	-	-	-	-	7.00
Face and neck (A_ap_ = 1.25)	-	-	-	-	7.50	-	-	-	3.75	-
Hand (A_ap_ = 1.00)	-	9.00	12.00	-	-	1.00	-	-	-	-
C_i_ [n·g^−1^]	2	9	12	3	6	1	4	2	3	7

**Table 8:** Dermal plastic absorption values for treatment cream samples.

Body Area	Dermal Plastic Absorption [n·day^–1^]								
	TC_1_	TC_2_	TC_3_	TC_4_	TC_6_	TC_7_	TC_8_	TC_9_	TC_10_
Scalp (A_ap_ = 1.50)	-	9.00	-	-	-	-	-	15.00	-
Face and neck (A_ap_ = 1.25)	-	7.50	8.75	8.75	-	11.25	-	12.50	-
Hand (front, back, and fingers) (A_ap_ = 0.50)	-	3.00	3.50	3.50	-	4.50	-	5.00	-
Arm (A_ap_ = 1.50)	-	9.00	-	-	-	-	-	15.00	-
Elbows (A_ap_ = 0.50)	1.00	3.00	3.50	3.50	-	4.50	-	5.00	-
Foot (A_ap_ = 0.75)	-	4.50	5.25	-	-	6.75	-	7.50	-
Knees (A_ap_ = 0.50)	1.00	3.00	3.50	-	-	4.50	-	5.00	-
Entire leg (A_ap_ = 4.00)	8.00	24.00	-	-	24.00	-	8.00	40.00	-
Trunk (A_ap_ = 7.00)	-	42.00	-	-	42.00	-	14.00	70.00	-
Entire arm (A_ap_ = 2.00)	-	12.00	-	-	12.00	-	4.00	20.00	-
Buttocks (A_ap_ = 2.50)	-	-	-	-	-	-	-	-	25.00
C_i_ [n·g^−1^]	2	6	7	7	6	9	2	10	10

## Data Availability

The original contributions presented in the study are included in the article and Supplementary Materials; further inquiries can be directed to the corresponding author.

## Supplementary Materials

The following supporting information can be downloaded at https://www.mdpi.com/article/10.3390/jox16010037/s1. Figure S1: Representative microparticles from skincare cream samples (different sizes, colors, and forms of fragments and fibers); Figure S2: Representative microparticles from treatment cream samples (different sizes, colors, and forms of fragments and fibers); Table S1: Information on respondents’ identification and frequency of use of skincare and treatment creams, as well as general opinions on microplastic pollution; Table S2: Types and ingredients of analyzed skincare/treatment creams according to the brand labels; Table S3: Morphological and chemical composition of microplastics in skincare cream samples; Table S4: Morphological and chemical composition of microplastics in treatment cream samples; Table S5. Risk to human health caused by dermal absorption of skincare creams contaminated with microplastics; Table S6. Risk to human health caused by dermal absorption of treatment creams contaminated with microplastics; Table S7. Estimated annual dermal absorption for microplastics identified in skincare creams; Table S8. Estimated annual dermal absorption for microplastics identified in treatment creams.
