# Supplementary material for: Identification, Quantification, and Characterization of Microplastics in Skincare and Treatment Creams: A Potential Health Concern Related to the Exposure Pathway
Source: J Xenobiot. 2026 Feb 22;16(1):37. doi: 10.3390/jox16010037 (PMC12941705; doi:10.3390/jox16010037)
Supplement: Supplementary file 1 [file jox-16-00037-s001.zip › jox-4146527-supplementary.pdf]

Identification, Quantification, and Characterization of Microplastics in Skincare and Treatment Creams: A Potential Health Concern Related to the Exposure Pathway

Raluca Maria Stirbescu [1], Cristiana Radulescu [2,3,4,*], Raluca Maria Bucur (Popa) [3], Andreea Laura Banica [1,3], Ioan Alin Bucurica [1] and Ioana Daniela Dulama [1]

[1] Institute of Multidisciplinary Research for Science and Technology, Valahia University of Targoviste, 130004 Targoviste, Romania; stirbescu.raluca@icstm.ro (R.M.S.); banica.andreea@icstm.ro (A.L.B.); bucurica_alin@icstm.ro (I.A.B.); dulama.ioana@icstm.ro (I.D.D.)
[2] Faculty of Sciences and Arts, Valahia University of Targoviste, 13 Sinaia Alley, 130004 Targoviste, Romania
[3] Doctoral School Chemical Engineering and Biotechnology, National University of Science and Technology Politehnica of Bucharest, 060042 Bucharest, Romania; ralucamaria.bucur@gmail.com
[4] Academy of Romanian Scientists, 3 Ilfov, 050044 Bucharest, Romania
* Correspondence: cristiana.radulescu@valahia.ro

Table S1. Information on respondents' identification and frequency of use of skincare and treatment creams, as well as general opinions on microplastic pollution (n = 354).

Variable	N [%]	Variable	N [%]
**Gender**		**Level of knowledge of the term "microplastic"**	
**Female**	274 (77.40)	Unknown	34 (9.60)
**Male**	80 (22.60)	Less known	58 (16.38)
		Somewhat known	103 (29.10)
		Well-known	132 (37.28)
		Very well-known	27 (7.63)
**Age [years]**		**Level of concern about microplastics**	
**18-20**	58 (16.38)	Not at all worried	50 (14.12)
**21-29**	62 (17.51)	A little worried	31 (8.76)
**30-39**	84 (23.73)	Somewhat worried	81 (22.88)
**40-49**	75 (21.19)	Worried	137 (38.70)
**50-59**	55 (15.54)	Very worried	55 (15.54)
**> 60**	20 (5.65)	**Microplastics can contain and accumulate hazardous chemicals**	
**Monthly budget [RON]**		Strongly agree	67 (18.93)
**No income**	69 (19.49)	Agree	131 (37.01)

**< 3.000**	44 (12.43)	Neither agree nor disagree	76 (21.47)
**3.000-6.000**	199 (56.21)	Disagree	42 (11.86)
**6.000-9.000**	32 (9.04)	Strongly disagree	20 (5.65)
**> 9.000**	10 (2.82)	Don't know	18 (5.08)
**Education level**		**Microplastics are transported throughout the trophic chain**	
**High school**	157 (44.35)	Strongly agree	57 (16.10)
**Bachelor's degree**	75 (21.19)	Agree	138 (38.98)
**Master's degree**	113 (31.92)	Neither agree nor disagree	77 (21.75)
**PhD**	9 (2.54)	Disagree	42 (11.86)
**The most used cream category**		Strongly disagree	20 (5.65)
**Beauty**	37 (10.45)	Don't know	20 (5.65)
**Treatment**	51 (14.41)	**Microplastics absorb and transport other emerging contaminants**	
**Beauty/Treatment**	266 (75.14)	Strongly agree	56 (15.82)
**Reasons to use beauty cream**		Agree	139 (39.27)
**Hydration**	1 (2.70)	Neither agree nor disagree	77 (21.75)
**Hydration and skin protection**	13 (35.14)	Disagree	42 (11.86)
**Hydration and cleansing**	12 (32.43)	Strongly disagree	20 (5.65)
**Skin protection**	1 (2.70)	Don't know	20 (5.65)
**Daily routine and hygiene**	10 (27.03)	**Knowledge regarding the presence of microplastics in creams**	
**Reasons to use treatment cream**		Yes	153 (43.22)
**Treatment**	42 (82.35)	No	201 (56.78)
**Hydration and treatment**	4 (7.84)	**Concerns about the presence of microplastics in creams**	
**Hydration and skin protection**	5 (9.80)	Yes	309 (87.29)
**Reasons to use beauty and treatment cream**		No	45 (12.71)
**Hydration**	17 (6.40)	**The extent of use of creams that may contain microplastics**	
**Skin protection**	29 (10.90)	Yes	71 (20.06)
**Reduction of imperfections**	24 (9.02)	No	33 (9.32)
**Hydration and skin protection**	32 (12.03)	Don't know	250 (70.62)
**Hydration and treatment**	27 (10.15)	**Microplastics affect human health following exposure through ingestion, inhalation, and/or dermal contact**	
**Hydration and reduction of imperfections**	85 (31.95)	Yes	120 (33.90)
**Hydration, skin protection, and treatment**	52 (19.55)	No	106 (29.94)
**Frequency of use of beauty creams**		Don't know	128 (36.16)
**Daily**	181 (51.13)	**The degree of purchase of more expensive products that do not contain microplastics**	
**2-3 times a day**	82 (23.16)	Strongly agree	47 (13.28)
**Weekly**	2 (0.56)	Agree	143 (40.40)

**2-3 times a week**	27 (7.63)	Neither agree nor disagree	101 (28.53)
**Monthly**	5 (1.41)	Disagree	53 (14.98)
**2-3 times a month**	6 (1.70)	Strongly disagree	10 (2.82)
**Does not use**	51 (14.41)	**The cosmetics and hygiene products industry is a generator of microplastics**	
**Frequency of use of treatment creams**		Strongly agree	80 (22.60)
**Daily**	75 (21.20)	Agree	166 (46.90)
**Weekly**	10 (2.82)	Disagree	56 (15.82)
**2-3 times a month**	21 (5.93)	Strongly disagree	52 (14.68)
**If necessary**	210 (59.32)		
**Does not use**	38 (10.73)		

Table S2. Types and ingredients of analyzed skincare/treatment creams according to the brand labels.

Cream type	Assigned Code	Ingredients declared by the producer on the label	Formulation type	Gender/ Age	Therapeutic recommendations	Application areas
**Skincare**	C_1	Water, glycerin, cetearyl alcohol, isopropyl palmitate, ethylhexyl stearate, glyceryl stearate citrate, glyceryl stearate, phenoxyethanol, citric acid, parfum, sodium cetearyl sulfate, *Cocos nucifera* oil, propylene glycol, xanthan gum, ethylhexylglycerin, tetrasodium glutamate diacetate, *Cocos nucifera* fruit extract, lactic acid, sodium hydroxide, potassium sorbate, sodium benzoate	Vegan	Women and men/ Adults	For dry body skin in cool seasons	Whole body
	C_2	Water, glycerin, C15-19 alkane, C10-18 triglycerides, dipropylene glycol, glyceryl stearate citrate, *Helianthus annuus* (sunflower) seed wax, pentylene glycol, squalane, sucrose stearate, corn starch modified, *Cocos nucifera* (coconut) oil, maltooligosyl glucoside, glyceryl caprylate/caprate, sclerotium gum, glycyrrhetinic acid, hydrogenated starch hydrolysate, xanthan gum, 1-methylhydantoin-2-imide, phytosphingosine, sodium citrate, citric acid, mannitol, xylitol, o-cymen-5-ol, benzotriazolyl dodecyl p-cresol, rhamnose, tocopherol, *Helianthus annuus* (sunflower) seed oil	Vegan	Women and men/ Adults	For atopic dermatitis	Hands
	C_3	Water, caprylic/capric triglyceride, glycerin, cetearyl alcohol, triolein, methylpropanediol, *Argania spinosa* kernel oil, potassium cetyl phosphate, isopropyl myristate, glyceryl stearate, dimethicone, polysorbate 60, dimethicone / vinyltrimethylsiloxysilicate crosspolymer, *Aloe barbadensis* leaf juice, *Ricinus communis* seed oil, hydrogenated castor oil, *Copernicia cerifera* wax, beeswax, *Vitis vinifera* seed oil, *Theobroma cacao* seed butter, tocopheryl acetate, panthenol, parfum, sodium polyacrylate, hydrogenated polydecene, trideceth-6, sodium gluconate,	Organic	Women / 30 -40 years	Restructuring night cream for face and neck	Hands

	caprylyl glycol, phenylpropanol, tocopherol, *Helianthus annuus* seed oil, potassium sorbate, sodium benzoate, citric acid, phenoxyethanol				
C_4	Water, cocoglycerides, glycerin, C13-15 alkane, isoamyl laurate, sodium acrylates copolymer, arachidyl alcohol, betaine, sodium hyaluronate, 1,2-hexanediol, allantoin, alpha-glucan oligosaccharide, arachidyl glucoside, behenyl alcohol, bisabolol, caprylyl glycol, cetearyl alcohol, citric acid, fructose, glucose, glyceryl stearate, *Helianthus annuus* seed oil, lactobacillus ferment, lecithin, maltose, panthenol, polyoxyethylene 100 stearate (PEG-100 stearate), pentylene glycol, phospholipids, sodium chloride, sodium citrate, sodium hydroxide, sodium lactate, sodium pyrrolidone carboxylic acid (sodium PCA), sphingolipids, *Spirulina platensis* extract, tetrasodium glutamate diacetate, tocopherol, tocopheryl acetate, trehalose, urea, xanthan gum, hydroxyacetophenone, p-anisic acid.	Organic	Women / 30-40 years	Restructuring day cream for face and neck	Face
C_5	Water, glycerin, squalane, caprylic/capric triglyceride, cetearyl alcohol, glyceryl stearate, ceteareth-20, glucosyl waxmide, pentylene glycol, sodium hyaluronate, phenoxyethanol, ethylhexylglycerin, calcium pantothenate, xanthan gum, urea, caprylyl glycol, magnesium chloride, potassium chloride, potassium lactate, magnesium lactate, sodium citrate, glucose, citric acid, dipalmitoyl hydroxyproline, tocopherol acetate, sodium polyacrylate, hydrogenated polydecene, trideceth-6, *Butyrospermum parkii* butter, parfum, butylated hydroxyanisole (BHA), tetrasodium EDTA (ethylenediaminetetraacetic acid salt), sodium hydroxide	Organic	Men/ Adults	Daily skin treatment for men around the age of 30	Face and neck
C_6	Water, glycerin, butylene glycol, isononyl isononanoate, dimethicone, citrus limon peel extract, *Zea mays* starch, trehalose, sodium	Organic	Women and men / Adults	Night cream for face and neck	Hands

	hyaluronate, ethylhexylglycerin, hydrolyzed hyaluronic acid, dimethicone crosspolymer, magnesium aspartate, caprylyl glycol, zinc gluconate, copper gluconate, isohexadecane, hydrogenated palm glycerides, dimethiconol, sorbitan isostearate, potassium cetyl phosphate, polysorbate 60, sodium laureth sulfate, acrylates/beheneth-25 methacrylate copolymer, hydroxyethyl acrylate/sodium acryloyldimethyl taurate copolymer, disodium EDTA (ethylenediaminetetraacetic acid salt), sodium hydroxide, tocopherol, sodium metabisulfite, phenoxyethanol, fragrance				
C_7	Water, glycerin, paraffinum liquidum, cetearyl alcohol, glyceryl stearate, *Prunus amygdalus* dulcis oil, panthenol, cetyl palmitate, dimethicone, carbomer, sodium hydroxide, phenoxyethanol, fragrance	Organic, Cruelty-free	Baby	Soothing and moisturizing effect.	Whole body
C_8	Water, glycerin, cetyl alcohol, glyceryl stearate, hydrogenated coco-glycerides, stearyl alcohol, microcrystalline wax, palmitic acid, stearic acid, tocopheryl acetate, sodium carbomer, myristic acid, arachidic acid, oleic acid, dimethicone, phenoxyethanol, methylparaben, geraniol, paraffinum liquid, fragrance	Organic	Men/ Adults	Prevents dryness and provides the necessary hydration of the men's skin	Face, neck, and hands
C_9	Water, glycerin, isopropyl stearate, cetyl alcohol, distarch phosphate, tocopheryl acetate, *Chamomilla recutita* flower extract, *Hamamelis virginiana* bark/leaf extract, maltodextrin, glyceryl stearate, palmitic acid, stearic acid, myristic acid, arachidic acid, oleic acid, caprylic/capric triglyceride, sodium hydroxide, dimethicone, carbomer, acrylates/C10-30 alkyl acrylate crosspolymer, phenoxyethanol, caprylyl glycol, butylated hydroxyanisole (BHA), linalool, alpha-isomethyl ionone, coumarin, geraniol, fragrance	Organic	Men/ Adults	For men's facial skin, prevent burning sensation, itching, "tight skin", micro-cuts, and red spots	Face and neck
C_{10}	Shea butter, cold-pressed oils (sweet almond, macadamia, brown rice, carrot, sea buckthorn,	Natural	Women and men/	Antioxidant, regenerating, antiaging effects	Face

		squalane, evening primrose, lamb's tongue, sesame, wheat germ, abyssinian), argan, Meadowfoam estolides (natural ceramides), colloidal silver, colloidal gold, colloidal platinum		Adults		
Treatment	TC_1	Purified water, *Arnica montana* (tincture), Carbomer 980, sodium hydroxide, 15%, ethanol 96% (v/v)	Natural, homeopathic	Adults and children aged over 1 year	Local treatment of benign trauma in the absence of wounds; recommendations for hematomas, sprains, muscle strains, joint dislocations, and pain.	Elbows, knees, and the entire leg
	TC_2	Water, paraffinum liquid, cetyl alcohol, glyceryl stearate, phenoxyethanol, sorbitol, caprylic/capric triglyceride, glycine soja oil, ceareareth-2, ceteareth-20, cetearyl alcohol, cetyl palmitate, acrylates/C10-30 aky acrylate crosspolymer, ethylhexylglycerin, *Calendula officinalis* flower extract, sodium hydroxide, *Aloe barbadensis* leaf juice powder, *Daucus carota satia* root extract, beta-carotene, tocopherol, limonene, citronellol, fragrance	Non-organic	Adults and children	Recommendation for sensitive skin: anti-inflammatory and antibacterial effect	Scalp, face and neck, hand (front, back, and fingers), arm, elbows, foot (top, sole, and toes), knees, entire leg, trunk, and entire arm and hand
	TC_3	Water, ethylhexyl palmitate, propylene glycol dicaprylate/dicaprate, polyethylene glycol ester of hyaluronic acid (PEG-8), propylene glycol, hydrogenated palm kernel oil, glyceryl stearate, polyoxyethylene 100 stearate (PEG-100 stearate), cetyl alcohol, *Mimosa tenuiflora* bark extract, myreth-3 myristate, carbomer, methylparaben, propylparaben, O-cymen-5-Ol, sodium hydroxide, 2-bromo-2-nitropropane-1,3-diol, *Helianthus annuus* (sunflower) seed oil, tocopherol, citric acid, potassium sorbate, sodium benzoate	Non-organic	Adults and children	Recommendation for damaged skin from small wounds and burns: repairing and healing effect	Face and neck, hand (front, back, and fingers), elbows, foot (top, sole, and toes), knees, and entire leg
	TC_4	Petrolatum, water, lanolin, paraffinum liquid, glyceryl stearate, white wax, *Hypericum perforatum* extract, *Calendula officinalis* flower extract, *Symphytum officinale* root extract, cetyl alcohol, *Plantago lanceolata* leaf extract, cholesterol,	Natural	Without restrictions	Recommendation for dry skin (xerosis, xeroderma); anti-inflammatory, healing action, stimulates	Face and neck, hand (front, back, and fingers), and elbows

	Chamomilla recutita flower extract, butylated hydroxyanisole (BHA)			the re-epithelialization process of tissues	
TC_5	Water, glycerin, sorbitol, hydroxyethylcellulose, *Plantago lanceolata* leaf extract, *Chamomilla recutita* flower extracts, sodium benzoate, chlorhexidine digluconate, sodium saccharin	Natural	Adults and children	Anti-inflammatory, analgesic, and healing effects	Hand (front, back, and fingers), arm, elbows, foot (top, sole, and toes), knees, entire leg, trunk, and entire arm and hand
TC_6	Water, glycerin, polyethylene glycol ester of hyaluronic acid (PEG-8), caprylyl glycol, carbomer, sodium hydroxide, fragrance, colorants, preservatives	Non-organic	Kids over 6 months	Soothing, cooling, restores the epithelial layer of the skin, against hives	Entire leg, trunk, and entire arm and hand
TC_7	Propolis, beeswax, *Helianthus annuus* seed wax (*Helianthus annuus* (sunflower) seed wax)	Natural	Adults and children	Recommendation for wounds, burns, eczema, herpes, acne, and boils; healing effect	Hand (front, back, and fingers), elbows, foot (top, sole, and toes), and knees
TC_8	Fir resin, propolis, beeswax, *Helianthus annuus* seed wax (*Helianthus annuus* (sunflower) seed wax)	Natural	Women and men/ Adults	Recommendations for varicose veins, varicose ulcer, shingles, and cellulite	Entire leg, trunk, and entire arm, and hand
TC_9	Petrolatum, water, lanolin, white wax, *Chelidonum majus* extract	Natural	Adults and children aged over 1 year	Recommendation for warts, herpes, psoriasis, wounds	Scalp, face and neck, hand (front, back, and fingers), arm, elbows, foot (top, sole, and toes), knees, entire leg, trunk, and entire arm and hand
TC_{10}	Water, paraffinum liquid, zinc oxide, paraffin, lanolin, ozokerite, sorbitan sesquioleate, benzyl benzoate, synthetic beeswax, benzyl alcohol, propylene glycol, benzyl cinnamate, parfum, linalyl acetate, butylated hydroxyanisole (BHA), citric acid, butylated hydroxytoluene (BHT), camphor, eucalyptol	Non-organic	Adults and children	Recommendation for inflamed skin: soothing and antiseptic effect	Buttocks

TC_{11}	Thermal water, DI-C12-13 alkyl malate, polymethyl methacrylate, dicaprylyl ether, tapioca starch, glycolic acid, sodium hydroxide, diglycerin, glycerin, malic acid, steareth-2, steareth-21, lactic acid, pentaerythrityl distearate, butylene glycol, acrylates/C10-30 alkyl acrylate crosspolymer, jojoba esters, dimethicone, glyceryl stearate, propanedionl, tocopheryl acetate, *Helianthus annuus* seed wax (*Helianthus annuus* (sunflower) seed wax) - piroctone olamine, xanthan gum, pentylene glycol, phytosphingosine, *Lens esculenta* (lentil) seed extract, polymethylsilsequioxane, salicylic acid, zinc lactate, asiaticoside, *Acacia decurrens* flower wax (*Acacia decurrens* flower wax), polyglycerin-3, *Glycyrrhiza inflata* root extract, sodium benzoate, rhamnose, glucose, glucuronic acid, tocopherol	Non-organic	Women and men/ Adults	Soothing cream recommended for deep hydration and immediate soothing of skin weakened by aggressive acne treatments	Scalp, face and neck, entire arm, and trunk

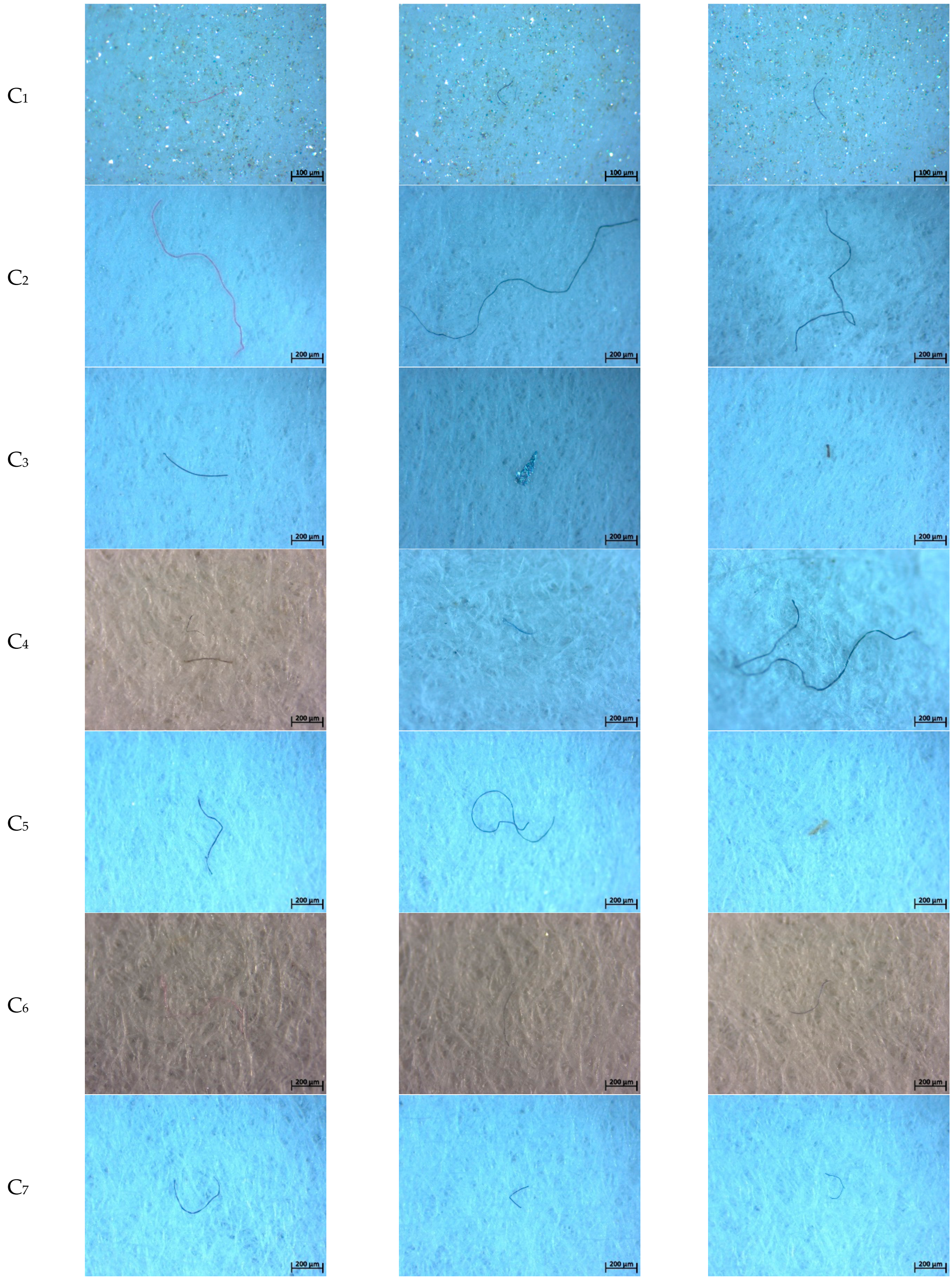
C1
C2
C3
C4
C5
C6
C7
100 µm
200 µm

C8

C9

C10

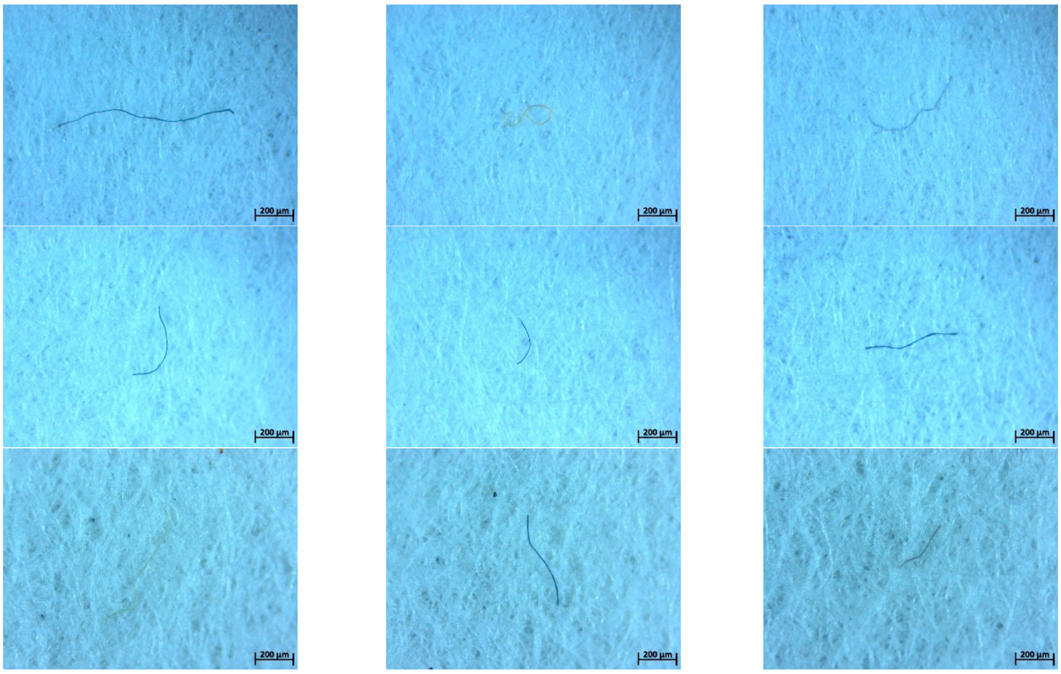

Figure S1. Representative microparticles from skincare cream samples (different sizes, colors, and forms of fragments and fibers).

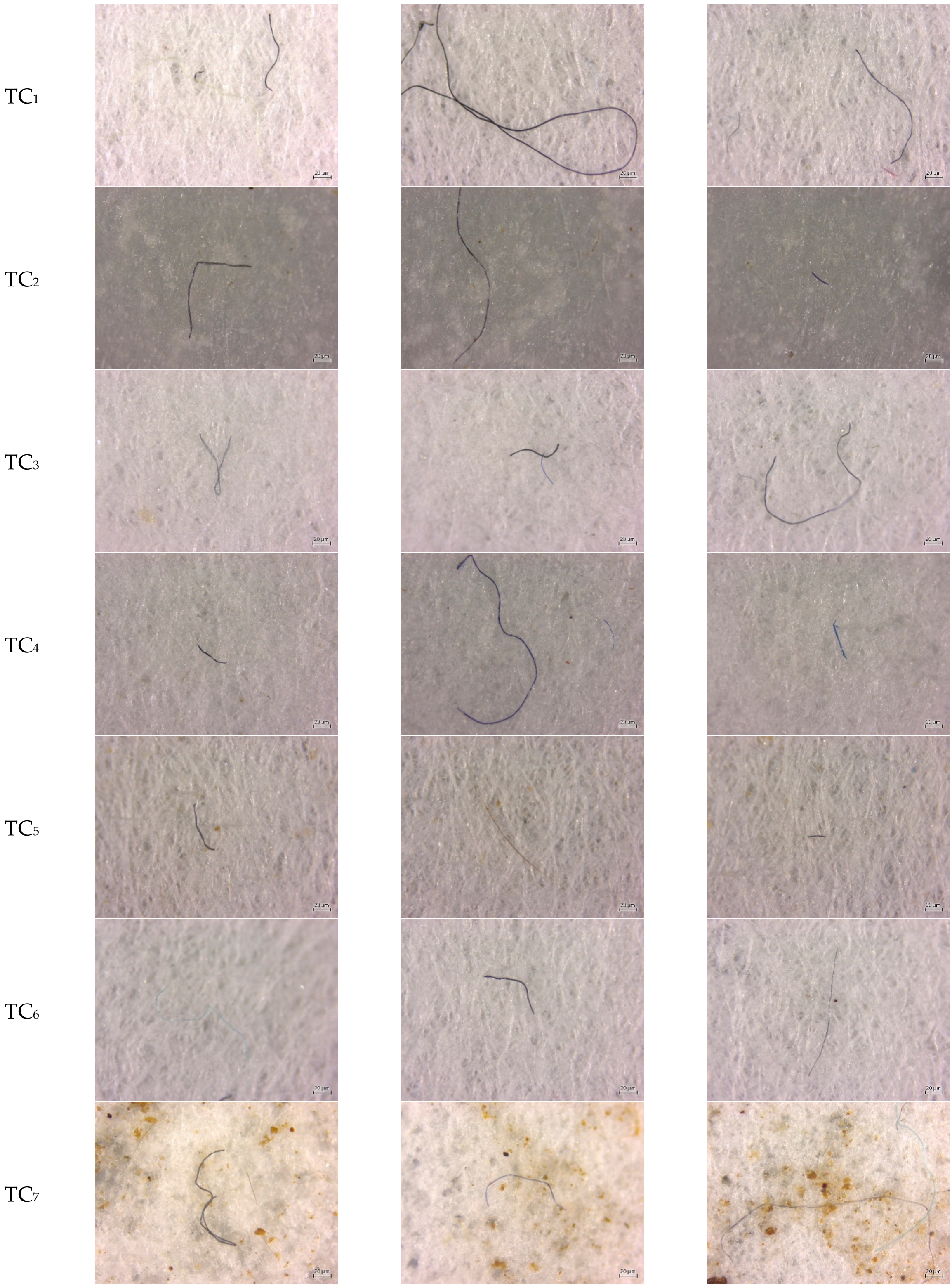
TC_1
TC_2
TC_3
TC_4
TC_5
TC_6
TC_7

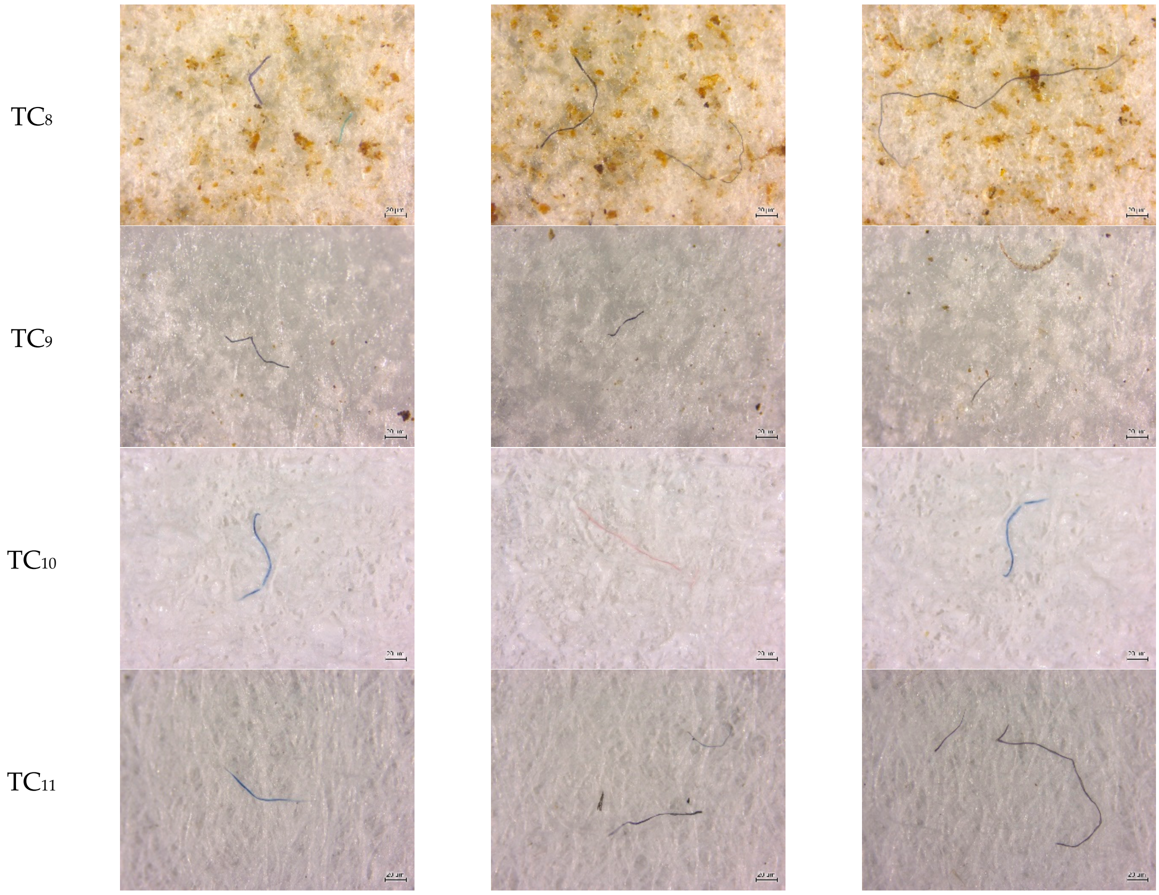

Figure S2. Representative microparticles from treatment cream samples (different sizes, colors, and forms of fragments and fibers).

Table S3. Morphological and chemical composition of microplastics in skincare cream samples.

Sample code	Micro-FTIR image	Composition of microplastics							Morphology	
		PE	PU	PES	PMMA	PA	Cotton	Cellulose acetate	Shape	Size [µm]
**$C_{1.1}$**	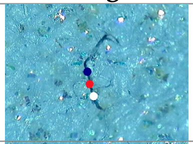	nd	nd	nd	40	nd	60	nd	Fiber	425.13
**$C_{1.2}$**	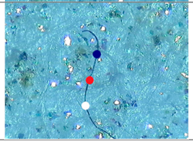	nd	10	40	nd	nd	nd	50	Fiber	> 584.34
**$C_{2.1}$**	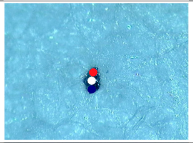	92	8	nd	nd	nd	nd	nd	Fragment irregular, blue	131.31x98.88
**$C_{2.2}$**	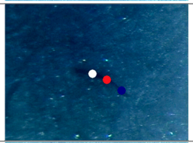	nd	nd	nd	40	nd	60	nd	Fiber	302.22
**$C_{2.3}$**	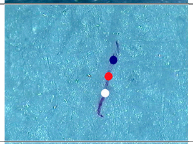	nd	5	nd	nd	nd	95	nd	Fiber	384.88
**$C_{2.4}$**	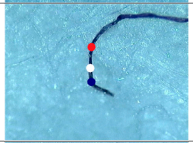	nd	50	nd	nd	nd	50	nd	Fiber	> 970.31
**$C_{2.5}$**	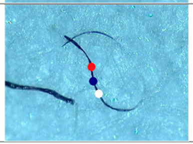	nd	5	nd	nd	nd	95	nd	Fiber	996.29

Sample code	Micro-FTIR image	Composition of microplastics							Morphology	
		PE	PU	PES	PMMA	PA	Cotton	Cellulose acetate	Shape	Size [µm]
**C$_{2.6}$**	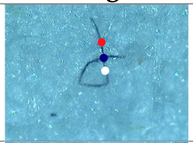	100	nd	nd	nd	nd	nd	nd	Fiber	1797.12
**C$_{2.7}$**	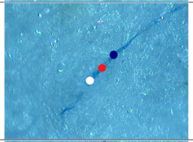	nd	nd	nd	40	nd	60	nd	Fiber	> 842.31
**C$_{2.8}$**	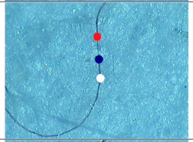	nd	5	nd	nd	nd	95	nd	Fiber	> 1093.12
**C$_{2.9}$**	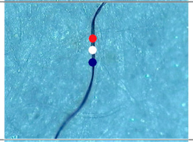	nd	5	nd	nd	nd	95	nd	Fiber	> 775.3
**C$_{3.1}$**	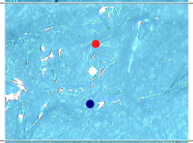	100	nd	nd	nd	nd	nd	nd	Fragment irregular, elongated, translucent	936.93x548.10
**C$_{3.2}$**	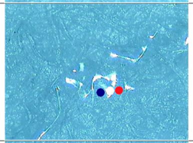	100	nd	nd	nd	nd	nd	nd	Fragment irregular, elongated, translucent	1059.26x247.80
**C$_{3.3}$**	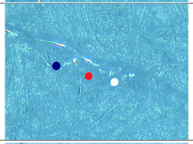	100	nd	nd	nd	nd	nd	nd	Fragment irregular, elongated, translucent	1020.50x185.19
**C$_{3.4}$**	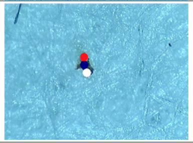	nd	nd	nd	40	nd	nd	60	Fragment irregular, white	130.21x86.58

Sample code	Micro-FTIR image	Composition of microplastics							Morphology	
		PE	PU	PES	PMMA	PA	Cotton	Cellulose acetate	Shape	Size [μm]
$C_{3.5}$	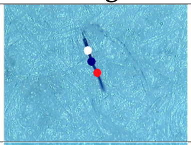	nd	nd	31	nd	nd	69	nd	Fiber	310.68
$C_{3.6}$	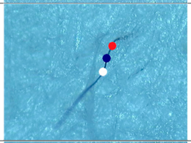	nd	nd	nd	40	nd	60	nd	Fiber	566.45
$C_{3.7}$	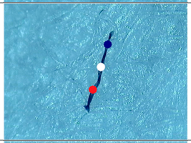	nd	nd	31	nd	nd	69	nd	Fiber	421.58
$C_{3.8}$	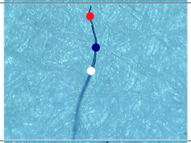	nd	nd	15	nd	nd	85	nd	Fiber	> 701.9
$C_{3.9}$	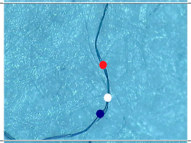	nd	nd	31	nd	nd	69	nd	Fiber	> 1138.89
$C_{3.10}$	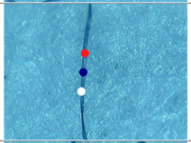	nd	nd	15	nd	nd	85	nd	Fiber	> 694.96
$C_{3.11}$	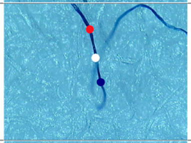	nd	nd	nd	40	nd	60	nd	Fiber	> 1087.24
$C_{3.12}$	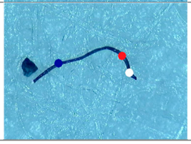	nd	nd	nd	100	nd	nd	nd	Fiber	540.92

Sample code	Micro-FTIR image	Composition of microplastics							Morphology	
		PE	PU	PES	PMMA	PA	Cotton	Cellulose acetate	Shape	Size [µm]
**$C_{4.1}$**	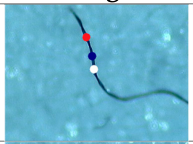	nd	nd	nd	15	nd	64	21	Fiber	> 920.65
**$C_{4.2}$**	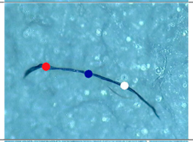	nd	nd	32	nd	nd	68	nd	Fiber	775.21
**$C_{4.3}$**	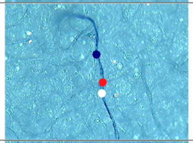	nd	nd	nd	50	nd	50	nd	Fiber	> 820.34
**$C_{5.1}$**	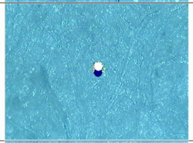	nd	nd	51	nd	nd	nd	49	Fragment irregular, elongated, translucent	70.91x112.11
**$C_{5.2}$**	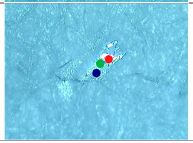	nd	nd	50	nd	nd	nd	50	Fragment irregular, elongated, translucent	351.27x172.42
**$C_{5.3}$**	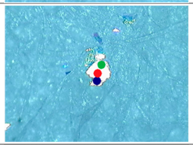	nd	10	40	nd	nd	nd	50	Fragment irregular, white	127.17x193.64
**$C_{5.4}$**	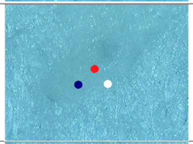	40	nd	nd	nd	10	nd	50	Fragment irregular, elongated, translucent	427.33x872.02
**$C_{5.5}$**	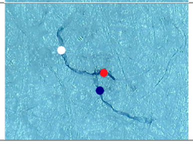	nd	nd	31	nd	nd	69	nd	Fiber	864.72

Sample code	Micro-FTIR image	Composition of microplastics							Morphology	
		PE	PU	PES	PMMA	PA	Cotton	Cellulose acetate	Shape	Size [μm]
**$C_{5.6}$**	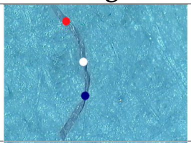	nd	nd	nd	21	nd	79	nd	Fiber	> 767.48
**$C_{6.1}$**	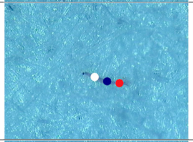	nd	nd	nd	6	11	70	13	Fiber	226.53
**$C_{7.1}$**	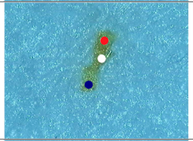	nd	nd	31	nd	nd	nd	69	Fragment irregular, elongated, slightly yellowish	326.13x99.63
**$C_{7.2}$**	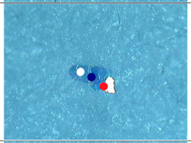	nd	nd	40	nd	nd	nd	60	Fragment irregular, translucent	260.69x132.30
**$C_{7.3}$**	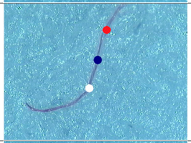	100	nd	nd	nd	nd	nd	nd	Fiber	> 835.19
**$C_{7.8}$**	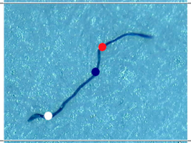	100	nd	nd	nd	nd	nd	nd	Fiber	799.04
**$C_{8.1}$**	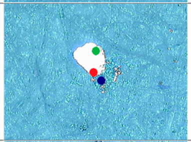	nd	nd	60	nd	nd	nd	40	Fragment irregular, white	169.55x140.80
**$C_{8.2}$**	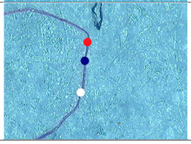	nd	nd	32	nd	nd	68	nd	Fiber	> 1112.21

Sample code	Micro-FTIR image	Composition of microplastics							Morphology	
		PE	PU	PES	PMMA	PA	Cotton	Cellulose acetate	Shape	Size [μm]
$C_{9.1}$	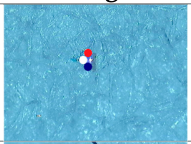	nd	nd	nd	6	11	13	70	Fragment irregular, elongated, translucent	126.00x112.34
$C_{9.2}$	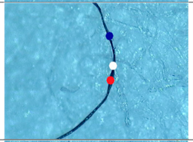	nd	nd	nd	21	nd	79	nd	Fiber	> 846.4
$C_{9.3}$	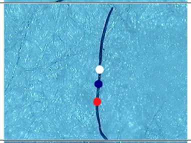	nd	nd	nd	40	nd	60	nd	Fiber	> 684.23
$C_{10.1}$	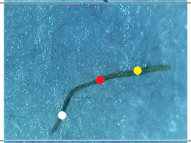	nd	nd	nd	40	nd	60	nd	Fiber	750.66
$C_{10.2}$	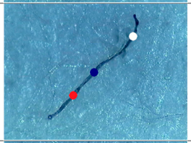	nd	nd	nd	21	nd	79	nd	Fiber	705.74
$C_{10.3}$	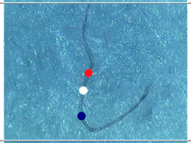	nd	nd	nd	15	nd	64	21	Fiber	1071.98
$C_{10.4}$	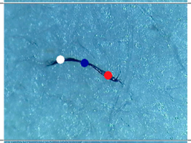	nd	nd	nd	21	nd	79	nd	Fiber	409.46
$C_{10.5}$	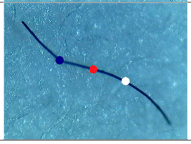	nd	nd	nd	100	nd	nd	nd	Fiber	954.19

Sample code	Micro-FTIR image	Composition of microplastics							Morphology	
		PE	PU	PES	PMMA	PA	Cotton	Cellulose acetate	Shape	Size [μm]
**$C_{10.6}$**	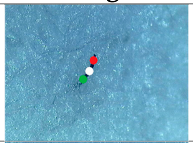	nd	nd	nd	40	nd	nd	60	Fragment irregular, elongated, translucent	183.79 x102.31
**$C_{10.7}$**	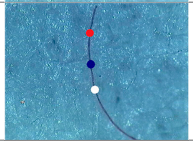	nd	nd	nd	40	nd	60	nd	Fiber	781.71

PES – polyester; PE – polyethylene; PU – polyurethane; PA – nylon; PMMA - poly(methyl methacrylate); nd – undetermined.

Table S4. Morphological and chemical composition of microplastics in treatment cream samples.

Sample code	Micro-FTIR image	Composition of microplastics							Morphology	
		Cotton	Cellulose acetate	PES	PE	PU	PA	PMMA	Shape	Size (LxW) [μm]
**$TC_{1.1}$**	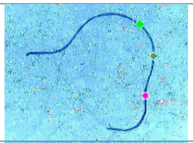	60	nd	nd	nd	nd	nd	40	Fiber	1,383.05
**$TC_{1.2}$**	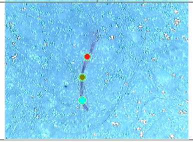	60	nd	nd	nd	nd	nd	40	Fiber	393.61
**$TC_{2.1}$**	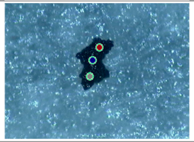	92	nd	nd	nd	8	nd	nd	Fragment irregular, black	257.89x 175.17
**$TC_{2.2}$**	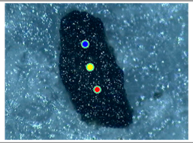	60	nd	nd	nd	nd	nd	40	Fragment irregular, black	625.27x 175.17
**$TC_{2.3}$**	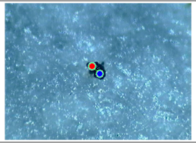	nd	50	nd	nd	nd	nd	50	Fragment irregular, black	112.55x 83.85
**$TC_{2.4}$**	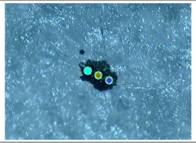	nd	50	nd	nd	nd	nd	50	Fragment irregular, black	206.92x 148.94
**$TC_{2.5}$**	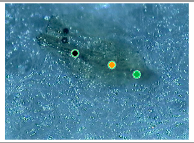	nd	50	nd	nd	nd	nd	50	Fragment irregular, elongated, translucent	623.60x 236.04

Sample code	Micro-FTIR image	Composition of microplastics							Morphology	
		Cotton	Cellulose acetate	PES	PE	PU	PA	PMMA	Shape	Size (LxW) [µm]
**TC$_{2.6}$**	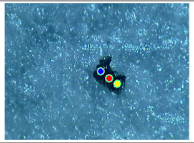	nd	70	nd	nd	nd	11	18	Fragment irregular, black	172.89x 118.90
**TC$_{3.1}$**	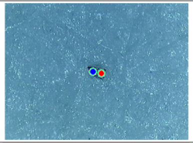	nd	79	nd	nd	nd	nd	21	Fragment irregular, black	99.05x 68.48
**TC$_{3.2}$**	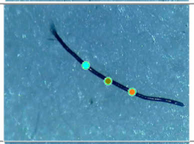	nd	50	nd	nd	nd	nd	50	Fiber	811.33
**TC$_{3.3}$**	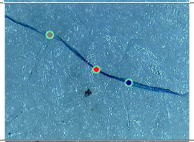	nd	50	nd	nd	nd	nd	50	Fiber	>1,027.04
**TC$_{3.4}$**	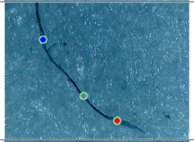	nd	50	nd	nd	nd	nd	50	Fiber	>903.33
**TC$_{3.5}$**	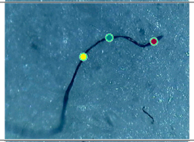	nd	50	nd	nd	nd	nd	50	Fiber	>927.90
**TC$_{3.6}$**	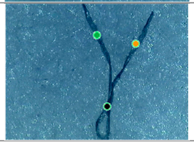	nd	50	nd	nd	nd	nd	50	Fiber	>1,485.6

Sample code	Micro-FTIR image	Composition of microplastics							Morphology	
		Cotton	Cellulose acetate	PES	PE	PU	PA	PMMA	Shape	Size (LxW) [µm]
**$TC_{3.7}$**	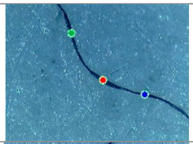	nd	60	nd	nd	nd	nd	40	Fiber	>945.61
**$TC_{4.1}$**	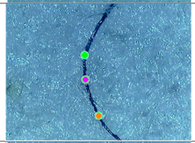	69	19	12	nd	nd	nd	nd	Fiber	>785.55
**$TC_{4.2}$**	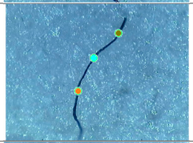	60	nd	nd	nd	nd	nd	40	Fiber	>720.48
**$TC_{4.3}$**	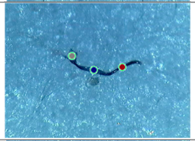	70	13	nd	nd	nd	11	6	Fiber	439.66
**$TC_{4.4}$**	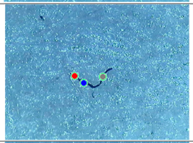	60	nd	nd	nd	nd	nd	40	Fiber	266.49
**$TC_{4.5}$**	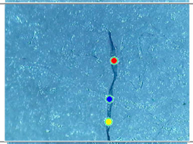	60	nd	nd	nd	nd	nd	40	Fiber	>571.77
**$TC_{4.6}$**	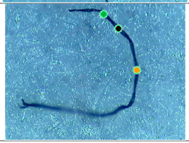	79	nd	nd	nd	nd	nd	21	Fiber	1,284.7
**$TC_{4.7}$**	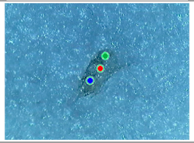	nd	50	nd	nd	nd	nd	50	Fragment irregular, elongated, translucent	269.65x 143.13

Sample code	Micro-FTIR image	Composition of microplastics							Morphology	
		Cotton	Cellulose acetate	PES	PE	PU	PA	PMMA	Shape	Size (LxW) [µm]
**$TC_{6.1}$**	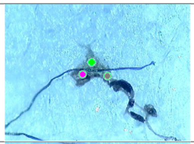	nd	50	nd	nd	nd	50	nd	Fragment irregular, translucent	162.64 x 133.51
**$TC_{6.2}$**	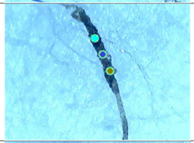	nd	50	nd	nd	nd	nd	50	Fiber	>740.15
**$TC_{6.3}$**	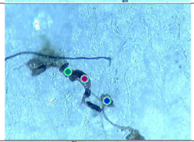	nd	nd	nd	100	nd	nd	nd	Fragment irregular, translucent	nd*
**$TC_{6.4}$**	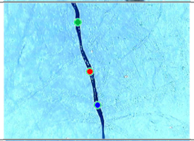	60	nd	nd	nd	nd	nd	40	Fiber	>717.57
**$TC_{6.5}$**	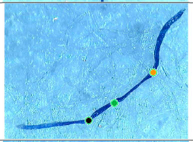	70	13	nd	nd	nd	11	6	Fiber	1,141.79
**$TC_{6.6}$**	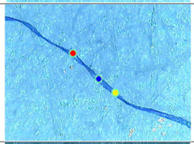	nd	nd	nd	100	nd	nd	nd	Fiber	>992.06
**$TC_{7.1}$**	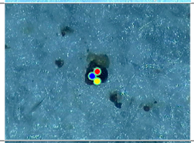	nd	92	nd	nd	8	nd	nd	Fragment irregular, translucent	123.03x 94.94
**$TC_{7.2}$**	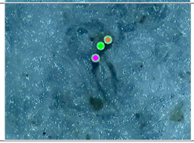	nd	50	nd	nd	nd	nd	50	Fragment irregular, translucent	189.79x 62.61

Sample code	Micro-FTIR image	Composition of microplastics							Morphology	
		Cotton	Cellulose acetate	PES	PE	PU	PA	PMMA	Shape	Size (LxW) [µm]
**TC$_{7.3}$**	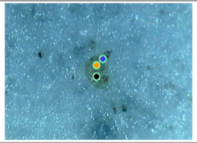	nd	65	nd	nd	nd	35	nd	Fragment irregular, elongated, translucent	181.41x 119.34
**TC$_{7.4}$**	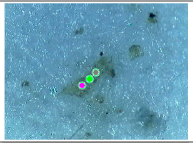	nd	nd	nd	100	nd	nd	nd	Fragment irregular, elongated, translucent	323.10x 83.77
**TC$_{7.5}$**	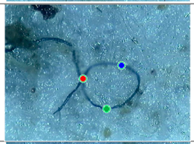	nd	nd	nd	100	nd	nd	nd	Fiber	1,576.48
**TC$_{7.6}$**	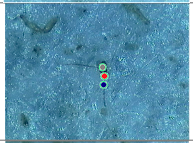	nd	50	nd	nd	nd	nd	50	Fragment irregular, translucent	>419.45
**TC$_{7.7}$**	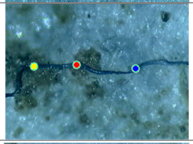	50	nd	nd	nd	nd	nd	50	Fiber	>1,066.68
**TC$_{7.8}$**	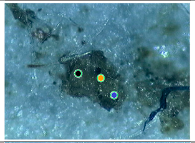	nd	70	nd	nd	nd	11	18	Fragment irregular, elongated, translucent	369.29x 259.43
**TC$_{7.9}$**	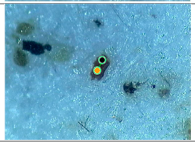	nd	50	nd	nd	nd	nd	50	Fragment irregular, translucent	138.34x 73.18

Sample code	Micro-FTIR image	Composition of microplastics							Morphology	
		Cotton	Cellulose acetate	PES	PE	PU	PA	PMMA	Shape	Size (LxW) [μm]
**TC$_{8.1}$**	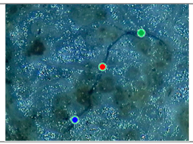	nd	nd	nd	100	nd	nd	nd	Fiber	849.03
**TC$_{8.2}$**	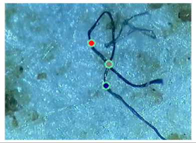	50	nd	nd	nd	nd	nd	50	Fiber	1,451.73
**TC$_{9.1}$**	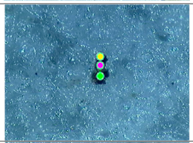	nd	nd	nd	100	nd	nd	nd	Fragment irregular, translucent	151.13x 97.82
**TC$_{9.2}$**	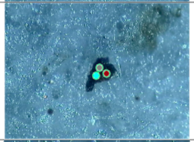	nd	nd	nd	100	nd	nd	nd	Fragment irregular, translucent	196.01x 106.13
**TC$_{9.3}$**	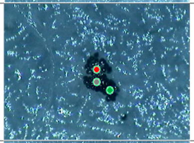	nd	60	nd	nd	nd	nd	40	Fragment irregular, black	242.48x 145.76
**TC$_{9.4}$**	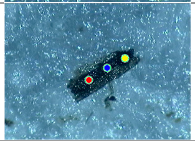	nd	50	nd	nd	nd	nd	50	Fragment irregular, black	373.77x 141.01
**TC$_{9.5}$**	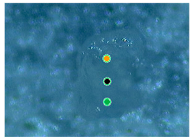	nd	95	nd	nd	8	nd	nd	Fragment irregular, elongated, translucent	431.94x 363.23

Sample code	Micro-FTIR image	Composition of microplastics							Morphology	
		Cotton	Cellulose acetate	PES	PE	PU	PA	PMMA	Shape	Size (LxW) [µm]
**$TC_{9.6}$**	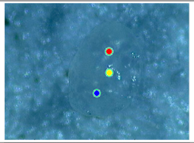	nd	65	nd	nd	nd	35	nd	Fragment irregular, elongated, translucent	503.33x 343.81
**$TC_{9.7}$**	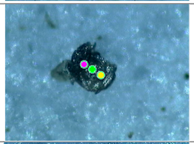	nd	50	nd	nd	nd	nd	50	Fragment irregular, translucent	237.69x 191.83
**$TC_{9.8}$**	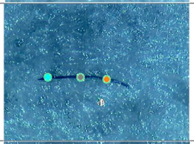	65	10	nd	nd	nd	25	nd	Fiber	453.93
**$TC_{9.9}$**	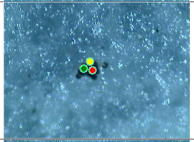	nd	nd	nd	100	nd	nd	nd	Fragment irregular, translucent	125.26x 104.81
**$TC_{9.10}$**	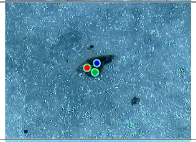	nd	90	nd	nd	10	nd	nd	Fragment irregular, translucent	193.63x 105.98
**$TC_{10.1}$**	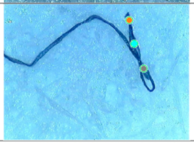	50	nd	50	nd	nd	nd	nd	Fiber	>1,835.88
**$TC_{10.2}$**	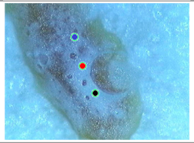	nd	nd	nd	100	nd	nd	nd	Fragment irregular, elongated, translucent	>790.76x 468.56

Sample code	Micro-FTIR image	Composition of microplastics							Morphology	
		Cotton	Cellulose acetate	PES	PE	PU	PA	PMMA	Shape	Size (LxW) [μm]
**$TC_{10.3}$**	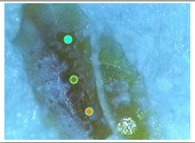	nd	nd	nd	100	nd	nd	nd	Fragment irregular, elongated, translucent	>760.77x 517.89
**$TC_{10.4}$**	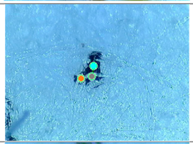	79	nd	nd	nd	nd	nd	21	Fragment irregular black	142.48x 124.64
**$TC_{10.5}$**	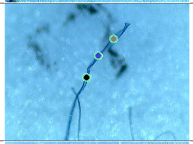	nd	nd	nd	100	nd	nd	nd	Fiber	>693.51
**$TC_{10.6}$**	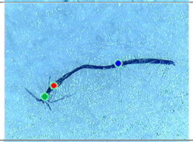	60	nd	nd	nd	nd	nd	40	Fiber	715.96
**$TC_{10.7}$**	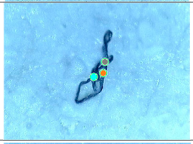	60	nd	nd	nd	nd	nd	40	Fiber	Nd*
**$TC_{10.8}$**	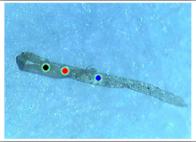	79	nd	nd	nd	nd	nd	21	Fragment irregular, elongated, translucent	1,304.64x145.47
**$TC_{10.9}$**	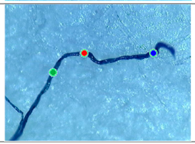	50	nd	nd	nd	nd	nd	50	Elongated fiber	>1,145.32

Sample code	Micro-FTIR image	Composition of microplastics							Morphology	
		Cotton	Cellulose acetate	PES	PE	PU	PA	PMMA	Shape	Size (LxW) [μm]
**TC$_{10.10}$**	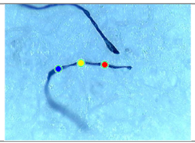	50	nd	nd	nd	nd	nd	50	Fiber	789.43

PES – polyester; PE – polyethylene; PU – polyurethane; PA – nylon; PMMA - poly(methyl methacrylate); Nd - underdetermined measurement of the fragment due to the presence of several fibers caught by the fragment; nd – undetermined.*

Table S5. Risk to human health caused by dermal absorption of skincare creams contaminated with microplastics.

**Bady Area**	**Risk to Human Health caused by Dermal Absorption***									
	**C_1**	**C_2**	**C_3**	**C_4**	**C_5**	**C_6**	**C_7**	**C_8**	**C_9**	**C_{10}**
Whole body	5,036,510.00	-	-	-	-	-	836,310.75	-	-	-
Face, neck, and hand	-	-	-	-	-	-	-	62,233.88	-	-
Face	-	-	-	70,846.50	-	-	-	-	-	40,402.43
Face and neck	-	-	-	-	244,180.00	-	-	-	29,190.41	-
Hand	-	221,912.00	33,117.20	-	-	6,676.00	-	-	-	-
**C_i [n·g^{-1}]**	**2**	**9**	**12**	**3**	**6**	**1**	**4**	**2**	**3**	**7**

RHHDA was calculated for the recommended application areas presented in Table S2, and the amounts of cream used per application, depending on the body surface area to be applied, are presented in Table 2.

Table S6. Risk to human health caused by dermal absorption of treatment creams contaminated with microplastics.

**Bady Area**	**Risk to Human Health caused by Dermal Absorption***								
	**TC_1**	**TC_2**	**TC_3**	**TC_4**	**TC_6**	**TC_7**	**TC_8**	**TC_9**	**TC_{10}**
Scalp	-	223,924.80	-	-	-	-	-	238,570.50	-
Face and Neck	-	186,604.00	56,701.96	58,774.79	-	-	-	198,808.75	-
Hand (front, back, and fingers)	-	74,641.60	22,680.79	23,509.92	-	22,032.60	-	79,523.50	-
Arm	-	223,924.80	-	-	-	-	-	238,570.50	-
Elbows	20,420.00	74,641.60	22,680.79	23,509.92	-	22,032.60	-	79,523.50	-
Foot	-	111,962.40	34,021.18	-	-	33,048.90	-	119,285.25	-
Knees	20,420.00	74,641.60	22,680.79	-	-	22,032.60	-	79,523.50	-
Entire leg	163,360.00	597,132.80	-	-	141,188.00	-	207,800.00	636,188.00	-
Trunk	-	1,044,982.40	-	-	247,079.00	-	363,650.00	1,113,329.00	-
Entire arm	-	298,566.40	-	-	70,594.00	-	103,900.00	318,094.00	-
Buttocks	-		-	-	-	-	-		97,192.50
**C_i [n·g^{-1}]**	**2**	**6**	**7**	**7**	**6**	**9**	**2**	**10**	**10**

RHHDA was calculated for the recommended application areas presented in Table S2, and the amounts of cream used per application, depending on the body surface area to be applied, are presented in Table 3.

Table S7. Estimated annual dermal absorption for microplastics identified in skincare creams.

Bady Area	Estimated Annual Dermal Absorption*									
	C_1	C_2	C_3	C_4	C_5	C_6	C_7	C_8	C_9	C_{10}
Whole body	16,242.50	-	-	-	-	-	32,485.00	-	-	-
Face, neck, and hand	-	-	-	-	-	-	-	1,642.50	-	-
Face	-	-	-	1,095.00	-	-	-	-	-	2,555.00
Face and neck	-	-	-	-	2,737.50	-	-	-	1,368.75	-
Hand	-	3,285.00	4,380.00	-	-	365.00	-	-	-	-
**C_i [$n \cdot g^{-1}$]**	**2**	**9**	**12**	**3**	**6**	**1**	**4**	**2**	**3**	**7**

**EADA was calculated for the recommended application areas presented in Table S2, and the amounts of cream used per application, depending on the body surface area to be applied, are presented in Table 2.*

Table S8. Estimated annual dermal absorption for microplastics identified in treatment creams.

Bady Area	Estimated Annual Dermal Absorption*								
	TC_1	TC_2	TC_3	TC_4	TC_6	TC_7	TC_8	TC_9	TC_{10}
Scalp	-	3,285.00	-	-	-	-	-	5,475.00	-
Face and Neck	-	2,737.50	3,193.75	3,193.75	-	-	-	4,562.50	-
Hand (front, back, and fingers)	-	1,095.00	1,277.50	1,277.50	-	1,642.50	-	1,825.00	-
Arm	-	3,285.00	-	-	-	-	-	5,475.00	-
Elbows	365.00	1,095.00	1,277.50	1,277.50	-	1,642.50	-	1,825.00	-
Foot	-	1,642.50	1,916.25	-	-	2,463.75	-	2,737.50	-
Knees	365.00	1,095.00	1,277.50	-	-	1,642.50	-	1,825.00	-
Entire leg	2,920.00	8,760.00	-	-	8,760.00	-	2,920.00	14,600.00	-
Trunk	-	15,330.00	-	-	15,330.00	-	5,110.00	25,550.00	-
Entire arm	-	4,380.00	-	-	4,380.00	-	1,460.00	7,300.00	-
Buttocks	-	-	-	-	-	-	-	-	9,125.00
**C_i [$n \cdot g^{-1}$]**	**2**	**6**	**7**	**7**	**6**	**9**	**2**	**10**	**10**

**EADA was calculated for the recommended application areas presented in Table S2, and the amounts of cream used per application, depending on the body surface area to be applied, are presented in Table 3.*
